# Embryonic vitamin D deficiency programs hematopoietic stem cells to induce type 2 diabetes

**DOI:** 10.1038/s41467-023-38849-z

**Published:** 2023-06-13

**Authors:** Jisu Oh, Amy E. Riek, Kevin T. Bauerle, Adriana Dusso, Kyle P. McNerney, Ruteja A. Barve, Isra Darwech, Jennifer E. Sprague, Clare Moynihan, Rong M. Zhang, Greta Kutz, Ting Wang, Xiaoyun Xing, Daofeng Li, Marguerite Mrad, Nicholas M. Wigge, Esmeralda Castelblanco, Alejandro Collin, Monika Bambouskova, Richard D. Head, Mark S. Sands, Carlos Bernal-Mizrachi

**Affiliations:** 1 Department of Medicine, Washington University School of Medicine, St. Louis, MO USA; 2 Department of Medicine, VA Medical Center, St. Louis, MO USA; 3 Department of Pediatrics, Washington University School of Medicine, St. Louis, MO USA; 4 Department of Genetics, Washington University School of Medicine, St. Louis, MO USA; 5 Instituto de Investigaciones en Ciencias de la Salud (INICSA), CONICET, Universidad Nacional de Córdoba, Córdoba, Argentina; 6 Department of Cell Biology and Physiology, Washington University School of Medicine, St. Louis, MO USA

**Keywords:** Diabetes, Calcium and vitamin D

## Abstract

Environmental factors may alter the fetal genome to cause metabolic diseases. It is unknown whether embryonic immune cell programming impacts the risk of type 2 diabetes in later life. We demonstrate that transplantation of fetal hematopoietic stem cells (HSCs) made vitamin D deficient in utero induce diabetes in vitamin D-sufficient mice. Vitamin D deficiency epigenetically suppresses *Jarid2* expression and activates the *Mef2*/*PGC1a* pathway in HSCs, which persists in recipient bone marrow, resulting in adipose macrophage infiltration. These macrophages secrete miR106-5p, which promotes adipose insulin resistance by repressing PIK3 catalytic and regulatory subunits and down-regulating AKT signaling. Vitamin D-deficient monocytes from human cord blood have comparable *Jarid2/Mef2/PGC1a* expression changes and secrete miR-106b-5p, causing adipocyte insulin resistance. These findings suggest that vitamin D deficiency during development has epigenetic consequences impacting the systemic metabolic milieu.

## Abstract (web summary)

Environmental conditions during pregnancy contribute to offspring metabolic disease. Here, the authors show that immune cells reprogrammed in utero by maternal vitamin D deficiency increase lifetime diabetes risk in the offspring and are sufficient to transplant diabetes.

## Introduction

Nearly 100 million Americans have either type 2 diabetes mellitus (T2DM) or prediabetes as a manifestation of insulin resistance (IR)^1^. Because rates of T2DM and its complications are increasing at such an alarming rate, America’s youth, for the first time in modern history, may have a shorter life expectancy than their parents^2^. Although chronic inflammation plays a crucial role in developing insulin resistance, the predisposing factors that alter immune cells are still unclear. Since the early 1980s, several historical cohort studies in diverse populations have provided evidence for the “developmental origins of adult disease hypothesis,” which posits that environmental factors in utero or during the early postnatal period program patterns of infant growth that result in increased susceptibility to IR and obesity later in life^3–5^. Therefore, identifying such factors and the tissues affected by the epigenetic program initiated in utero are key to developing patient treatment strategies and preventative therapies for future generations.

During embryogenesis, the genome undergoes a critical period of reprogramming in response to environmental stimuli^6^. While this process of genome-wide epigenetic reprogramming can provide an evolutionary benefit by facilitating rapid adaptation of the phenotype to the environment, it can also induce lifelong maladaptive changes, predisposing individuals to IR and obesity later in life^7,8^. Early studies examining the regulation of metabolic disease by the epigenome from diverse exposures such as maternal under- or overnutrition were focused on metabolically active tissues such as the liver, endocrine pancreas, and adipose tissue. However, even though immune cells might be a unifying mechanism causing metabolic disease, there is a lack of studies that identify the epigenetic signature(s) of immune cells programmed during embryogenesis that predisposes to adult onset of IR and diabetes.

Changes to chromatin structure and function during embryogenesis are critical in determining appropriate gene expression patterns that drive distinct cell lineages^9^. Epigenetic modifications such as DNA CpG methylation and covalent modifications of histones can alter gene expression, often by structural modifications in the absence of changes in DNA sequence^10^. Modulation of DNA methylases (DNMT) along with histone-modifying enzymes regulating chromatin function have been previously linked to IR^11^. Deleting the H3K9-specific demethylase Jhdm2a induces obesity, hyperlipidemia, and IR in mice by repressing adipose PPARα and UCP-1 expression^12^. Mice lacking DNMT3a are protected from diet-induced IR and glucose intolerance^13^, and pharmacological inhibition of DNMTs relieves DNMT1-mediated repression of adipose adiponectin expression, improving IR in obese mice^14^. These data suggest that genetic and pharmacologic inhibition of chromatin-opening enzymes promotes global metabolic dysregulation and IR^15^.

Interestingly, studies in monozygotic twins have suggested a direct correlation between global DNA methylation in peripheral blood leukocytes and the severity of IR, implicating the epigenetic modification of immune cells in the development of diabetes^16^. Moreover, epidemiological studies have demonstrated an association between inactivating mutations of the epigenetic enzyme TET2, which induces clonal myeloid and lymphoid expansion, and the development of T2DM and cardiovascular disease in humans^17,18^. In mice, a causal relationship between epigenetic immune cell dysfunction and metabolic disease has been illustrated by bone marrow transplantation from donor mice with inactivating mutations of TET2 to wild-type recipient mice, which was sufficient to induce macrophage adipose infiltration, pro-inflammatory cytokine IL-1β secretion, and obesity-related IR^19^. Thus, discovering environmental factor(s) regulating the enzymes controlling the chromatin state in immune cells during embryogenesis could be critical for preventing chronic inflammatory diseases such as diabetes.

In the U.S., 80% of pregnant African-American females and 60% of pregnant Caucasian females are vitamin D-deficient or insufficient^20^. Vitamin D deficiency [VD(-)] is associated with low birth weight and small for gestational age, processes which increase susceptibility to obesity, IR, and diabetes later in life^21–23^. Murine studies confirm that in utero vitamin D deficiency results in offspring systemic inflammation, hepatic steatosis, excess adiposity, and IR that persist despite vitamin D supplementation after birth, implying that vitamin D deficiency during gestation induces epigenetic programming^24–28^. However, the tissue(s) carrying the underlying cellular program to cause offspring IR has remained elusive.

Multiple studies strongly suggest a role for the vitamin D receptor (VDR) in hematopoiesis. VDR knockout mice have persistent changes in lymphocytic and myelocytic function and cytokine profiles, suggesting the importance of VDR signaling for immune cell programming during embryogenesis^29^. Vitamin D is known to regulate multiple components of the epigenetic machinery, though the net effects of this regulation are mixed depending upon the system studied^30^. Polycomb group (PcG) proteins, made up of the initiation complex PRC2 and maintenance complex PRC1, play an important role in maintaining transcriptional repression of target genes through chromatin modifications^31^. PRC2 requires Jarid2 for its repressive activity, and both affect the proliferative and self-renewal capacities of hematopoietic stem cells to influence their immune program ^32^. Prior studies have linked active vitamin D to the upregulation of murine macrophage *Jarid2* expression, but the effects of this interaction on the immune cell epigenome as it contributes to metabolic disease are unknown^30^.

Our previous studies indicated that macrophage-specific VDR deletion is sufficient to induce IR and hypertension by promoting a pro-inflammatory macrophage phenotype in metabolic tissues, suggesting that altered VDR signaling in immune cells during embryogenesis programs pro-inflammatory immune cells to cause IR in the offspring^28,33^. In this study, by using transplantation of fetal hematopoietic stem cells (HSCs) exposed to VD deficiency in utero into VD-sufficient mice, we identified an epigenetic program of a single, non-metabolically active tissue compartment that is mitotically stable and sufficient to induce type 2 diabetes.

## Results

### Hematopoietic stem cells from vitamin D-deficient embryos transplant insulin resistance

To determine whether the fetal immune cell program induced by VD deficiency is sufficient to cause IR in different mouse backgrounds, C57BL/6 and C57BL/6-LDLR^−/−^GFP^+/−^ (a model of diet-induced IR) female mice were fed a vitamin D-deficient or sufficient diet four weeks before pregnancy. We confirmed that the dams were vitamin D deficient at mid-gestation [25(OH)D 9 ± 3 vs. 35 ± 2 ng/mL]. There were no differences in dam or fetus weights, food intake, serum calcium, glucose, or lipids between VD(−) and VD(+) dams (Supplementary Fig. 1A–E). We isolated fetal (E13) liver HSCs from embryos obtained from VD(−) and VD(+) dams in the C57BL/6 and C57BL/6-LDLR^−/−^GFP^+/−^ background. VD(-) isolated fetal liver donor cells had a higher percentage of long-term (LT) and short-term (ST) HSCs, common myeloid progenitors (CMP), granulocyte-macrophage progenitors (GMP), megakaryocyte erythroid progenitors (MEP), and macrophage dendritic cell progenitors (MDP) compared to VD(+) isolated fetal liver donor cells (Supplementary Fig. 2A–D). Fetal liver cells were then transplanted into genotype-matched 8-week-old VD-sufficient mice in the C57BL/6 or C57BL/6-LDLR^−/−^ GFP^+/−^ background, respectively. Eight weeks after transplantation, both groups of primary recipients had similar weights, and 90% of peripheral blood cells were of donor origin. Furthermore, 98% of epididymal immune cells from the stromal vascular fraction (SVF) of recipients 30 weeks after transplantation were of donor origin (Supplementary Fig. 3A–E). Recipient BM cells of VD(−) HSCs demonstrated a pattern of immune cell progenitors similar to that of VD(−) donor HSCs with a higher percentage of LT- and ST-HSCs and myeloid progenitors compared to VD( + ) BM cells (Supplementary Fig. 3F).

**Fig. 1:**
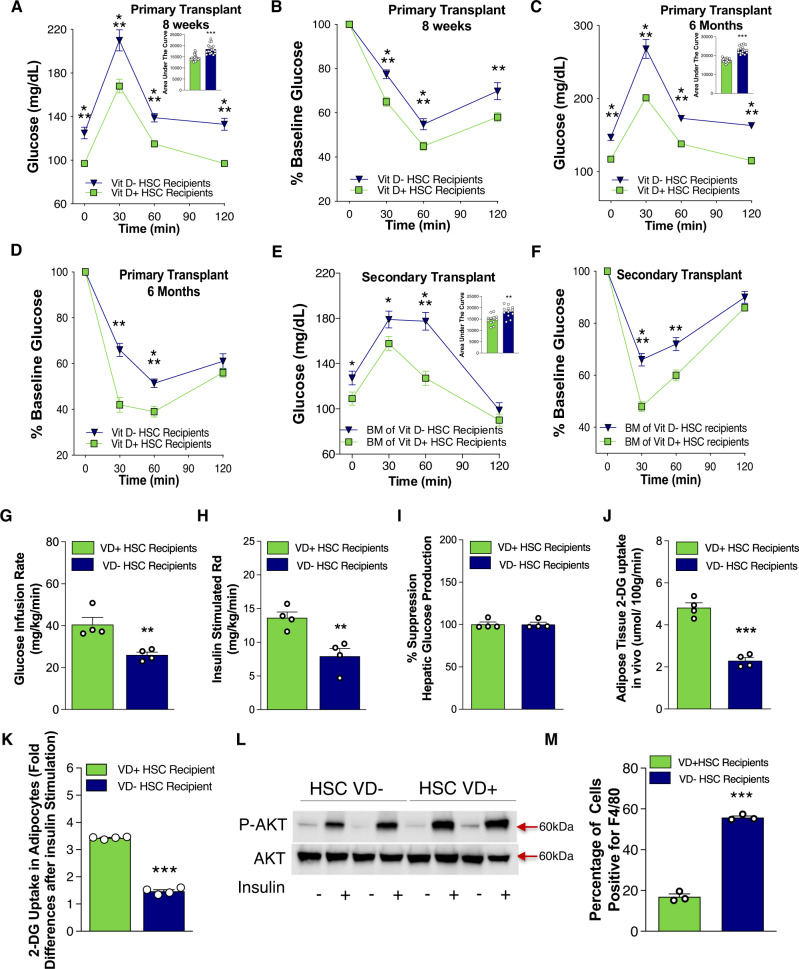
In utero VD deficiency reprograms HSCs to transfer IR. **A**–**J** Vitamin D-sufficient CD45.1^+^ C57BL6 mice were transplanted with VD(−) or VD( + ) FL-HSCs from CD45.2^+^ C57BL6 mice (primary; *n* = 20/group), then these primary recipients were used as BM transplant donors for vitamin D-sufficient mice (secondary; 20 transplanted mice/group). Glucose and insulin tolerance tests were performed at (**A**, **B**) 8 weeks VD(−)(*n* = 18, VD( + ) = 20 mice/group from two independent experiments) and **C**, **D** 6 months post-primary-transplant (VD(−) *n* = 15, VD(+) *n* = 16 mice) and **E, F** 8 weeks post-secondary-transplant (*n* = 12/group). The area under the curve is included on the glucose tolerance test insets. Hyperglycemic-euglycemic clamps were conducted in primary transplant recipients after 8 weeks (*n* = 4/group). Data are reported as **G** glucose infusion rate, **H** insulin-stimulated glucose disposal rate (Rd), **I** change in hepatic glucose production, and **J** insulin-stimulated 2-DG uptake in adipose tissue. **K**–**M** Primary eWAT was isolated from VD(−) or VD( + ) FL-HSC recipients. **K** Ex vivo insulin-stimulated 2-DG uptake (*n* = 4/group). **L** Western blot analysis of phospho- and total AKT levels following insulin stimulation. **M** Percentage of F4/80-positive cells by manually counting 15 light microscopy fields under ×20 objective per mouse (*n* = 3/group). Data presented as mean ± SEM. *P < 0.05; **P < 0.01; ***P < 0.001 vs. VD( + ) FL-HSCs recipients by two-tailed unpaired *t* test. Actual *P* values are shown in the source data file.

To examine the metabolic implications of both groups of recipient mice, we performed intraperitoneal glucose tolerance testing (GTT) and insulin tolerance testing (ITT). Mice of both sexes and genetic backgrounds receiving VD(−) HSCs demonstrated fasting hyperglycemia, impaired glucose tolerance, and IR, independent of the VD status of the recipient mice (Fig. 1A, B and Supplementary Fig. 4A, B). To determine if the IR phenotype was transmitted by multipotent VD(−) HSCs and preserved over time despite normal VD status in all recipients, we evaluated the IR phenotype in 6-month-old VD(+) primary recipients and also performed secondary transplants into VD(+) recipients using bone marrow from VD(+) primary recipients transplanted with VD(−) HSCs. At 6 months post-transplant, both primary and secondary recipients maintained a stable IR phenotype (Fig. 1C–F). LDLR deletion did not affect the IR phenotype induced by VD(−) HSCs in either primary or secondary recipients (Supplementary Fig. 4C–F). Transfer of VD(−) HSCs did not alter recipient insulin levels after glucose challenge (Supplementary Fig. 5A–C). These data confirm that in utero VD deficiency induces an HSC program that confers persistent IR in VD-sufficient primary and secondary transplant recipients.

Hyperinsulinemic-euglycemic clamping performed 8 weeks after the primary transplant showed that peripheral rather than hepatic IR was induced in C57BL6 VD(-) HSC recipients under conditions of vitamin D sufficiency (Fig. 1G–I). Insulin-stimulated uptake of 2-deoxyglucose (2-DG) at the end of the clamp identified perigonadal fat rather than the muscle as the primary insulin-resistant tissue (Fig. 1J and Supplementary Fig. 5D, E). These findings in epididymal white adipose tissue (eWAT) of VD(−) HSC recipients were supported ex vivo by a reduction in insulin-stimulated 2-DG uptake and phospho-AKT. The eWAT demonstrated increased immune cell infiltration/proliferation that was >99% donor-derived with a predominance of pro-inflammatory M1 macrophages with increased adhesion and migration (Fig. 1K–M and Supplementary Fig. 6A–F), suggesting that the eWAT macrophage population responsible for inducing IR was not an embryonic-derived from the host resident macrophages. We also analyzed immune cell infiltration in subcutaneous adipose tissue, liver, brown fat, and muscle and eWAT adipocyte size in recipients at 30 weeks post-primary transplant and identified no difference in immune cell number in these tissues or eWAT adipocyte size (Supplementary Figs. 6G and  7A–E). Since these findings implicated macrophage eWAT infiltration as the primary mechanism by which IR is induced in VD(−) HSC recipients, we limited our analysis of macrophage phenotype and function to those isolated from eWAT. Together, these results suggest that in utero VD deficiency induces an HSC program that increases epidydimal adipose macrophage infiltration/proliferation to cause IR.

### Fetal vitamin D deficiency represses Jarid2 expression

**Fig. 2:**
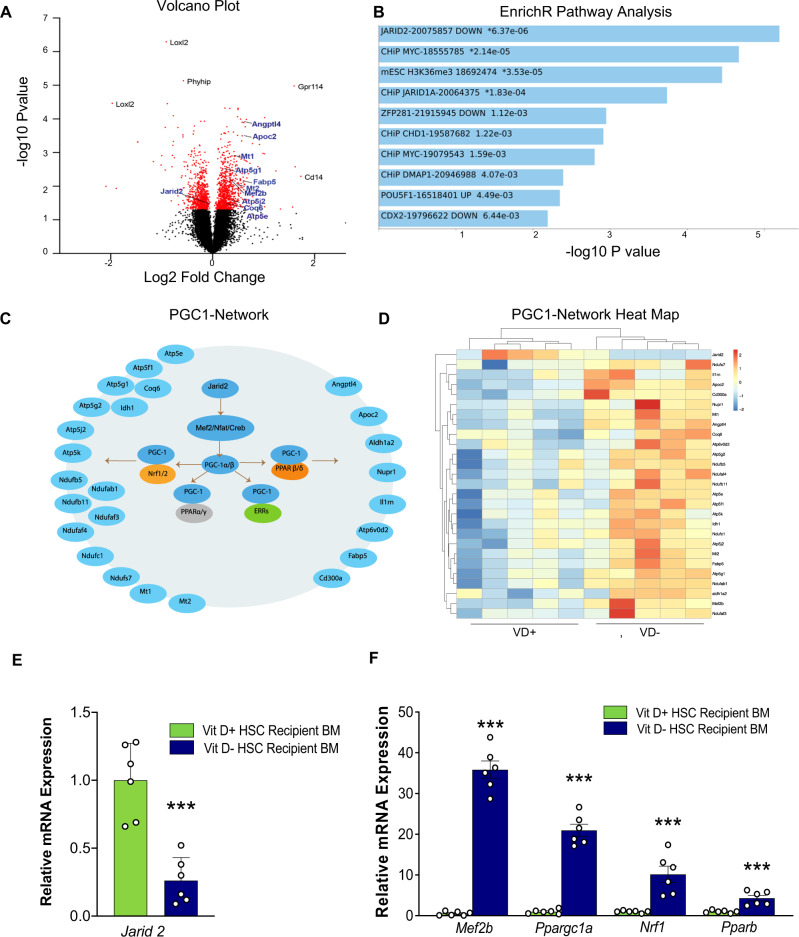
Top genes, networks, and pathways identified in transcriptome analysis of bone marrow from recipient mice transplanted with HSCs isolated from embryos from VD-sufficient and -deficient dams. VD(−) vs. VD( + ) FL-HSCs were transplanted into VD(+) mice, and global mRNA expression was evaluated by microarray in recipient BM cells at 16 weeks post-transplant. **A** Volcano plot showing top differentially expressed genes. Red dots indicate those array probes with *P* < 0.05. Black represents the non-significant probes. Genes of the Jarid2-PGC1-MEF2 pathway are highlighted in blue. *P* values for each probe were calculated using a two-tailed *t* test between five replicates in each condition. **B** Genes with significant changes were used for manual and automated pathway analysis. The figure shows EnrichR (PMID 23586463; 27141961; 33780170) top pathway hits from the ESCAPE database. Asterisks indicate those pathways that pass multiple testing corrections. EnrichR used Fisher’s exact test for *P* value calculation and Benjamini–Hochberg test for multiple testing corrections (**C**) Illustration of the Jarid2-MEF2-PGC1 network and the target genes that are differentially expressed in the array data. **D** Heatmap table showing normalized gene expression for Jarid2, MEF2-PGC1 target genes. Red indicates upregulation, and blue indicates downregulation. **E**, **F** Quantitative RT-PCR in FL-HSC transplant recipient BM to confirm expression changes in Jarid2 and PGC1α network-related genes (*n* = 6/group). Data presented as mean ± SEM. ***P < 0.001 vs. VD( + ) FL-HSCs recipients by two-tailed unpaired *t* test. Actual *P* values are shown in the source data file.

**Fig. 3:**
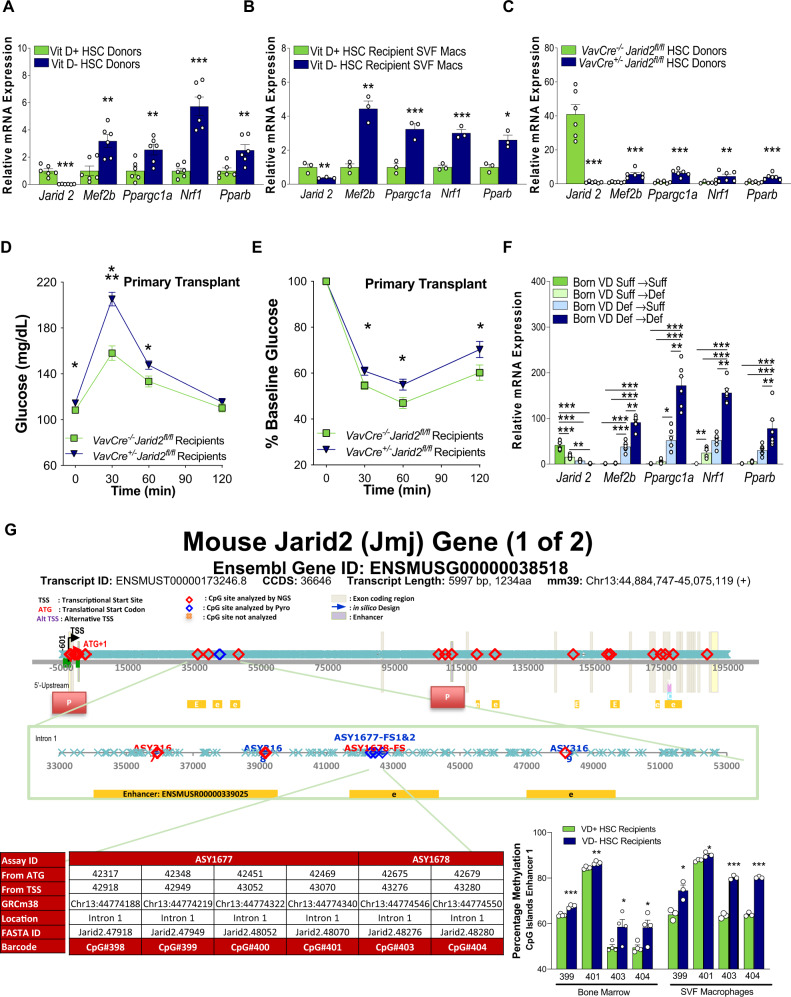
In utero VD deficiency epigenetically suppresses Jarid2 expression in macrophages, resulting in persistent *PGC1α* network upregulation. **A**, **B** Quantitative RT-PCR in donor FL-HSCs and SVF macrophages from FL-HSC transplant recipients (*n* = 6/group). **C** Quantitative RT-PCR in HSCs from mice with deletion of Jarid2 in HSC vs. controls (*n* = 12/group). **D**, **E** Glucose and insulin tolerance tests were performed at 8 weeks post-primary-transplant of HSC with deletion of Jarid2 or controls (*n* = 14/group) **F** Quantitative RT-PCR in macrophages to analyze the expression of Jarid2 and PGC1α network-related genes in mice born VD(−) or VD(+) then fed VD(−) or VD(+) diet postnatally (*n* = 6/group). **G** Targeted next-generation sequencing methylation assays were performed to interrogate the 69 CpG sites in the 5’ Upstream through 3’ UTR regions of the mouse *Jarid2* gene, including two promoter regions and several enhancers, in BM and SVF macrophages isolated from VD(−) and VD( + ) FL-HSCs recipients at 24 weeks (*n* = 4/group). The gene structure of mouse Jarid2 and the regions throughout the gene where the CpG methylation was interrogated are shown. The methylation assays highlighted in red demonstrate a significant difference (*P* < 0.05) between the two cohorts. The CpGs in these regions are shown in orange boxes. The % methylation increase and *P* values are reported. The gray boxes are those CpGs that have suggestive changes. Data presented as mean ± SEM. **P < 0.01; ***P < 0.001 by two-tailed unpaired *t* test except in (**D**) where *P < 0.05; **P < 0.01; ***P< 0.001 by one-way ANOVA followed by Tukey’s multiple comparison test. Actual *P* values are shown in the source data file.

To elucidate the HSC program associated with VD deficiency, we performed a multi-omic analysis of mRNA and miRNA expression. Transcriptome analysis showed consistent upregulation of 391 genes and downregulation of 657 genes in the BM of VD(−) vs. VD( + ) HSC transplant recipients at 8 weeks post-transplant (GEO: GSE158763) (Fig. 2A). Enrichment pathway analysis identified the Jarid2 pathway signature as the most significantly activated (Fig. 2B and Supplementary Table 1). Jarid2, a histone methyltransferase that is part of the polycomb repressive complex 2 (PRC) and critical for immune cell differentiation^31^, was downregulated in recipient BM and long-term BM HSCs of VD(−) HSCs, resulting in the expected activation of downstream genes involved in metabolic function, specifically myocyte enhancer factor 2 (Mef2) and its coactivator, peroxisome proliferator-activated receptor gamma coactivator 1-alpha (PGC1*α*) (Fig. 2C–F and Supplementary Fig. 8A)^34–36^. Moreover, alterations in the *Jarid2/Mef2/PGC1α* pathway were also present in total and long-term VD(−) HSCs donors (Fig. 3A and Supplementary Fig. 8B), recipient mouse eWAT adipose tissue macrophages (ATM) and peritoneal macrophages despite normal plasma VD levels in recipient mice (Fig. 3B and Supplementary Fig. 8C), suggesting that this genetic program activated in VD(−) HSCs persisted in immune cells when transplanted into VD-sufficient mice. These findings are consistent with previous evidence that VD stimulates *Jarid2* expression^30^. Indeed, we found that E13 fetal liver HSCs with the deletion of *Jarid2* expression from *Vav1Cre* + */−Jarid2fl/fl* mice have activated the *Mef2/PGC1a* pathway and induced fasting hyperglycemia, impaired glucose tolerance, and IR in wild-type recipients of VD(+) *Jarid2*−/− HSCs when compared to recipients *of fetal liver Jarid2* + */+* HSC, confirming the importance of the downregulation of HSC Jarid2 in programming immune cells to cause IR (Fig. 3C–E).

To determine if this immune cell program and the IR phenotype induced by VD deficiency in utero were transmitted through changes in the immune cell epigenome, pups exposed to VD deficiency in utero were maintained on a VD-deficient diet or transitioned to standard diet postnatally. Importantly, postnatal VD supplementation neither restored Jarid2 expression nor corrected the impaired glucose tolerance and IR induced by in utero VD deficiency, indicating that epigenetic modifications resulting from in utero VD deficiency are responsible for the observed changes in macrophage *Jarid2* expression that contribute to IR. We also observed that ongoing maintenance of VD-deficient pups on a VD-deficient diet resulted in a more severe IR phenotype, suggesting that maintenance of VD-deficiency postnatally can exacerbate the in utero VD-deficient phenotype (Fig. 3F and Supplementary Fig. 9). We hypothesized that the gene expression changes leading to stable reprogramming of the *Jarid2/Mef2/PGC1α* pathway were a consequence of changes in *Jarid2* methylation status. Therefore, we performed targeted next-generation sequencing (NGS) methylation assays to interrogate the DNA methylation status of 69 CpG sites in the 5’ upstream through 3’ UTR regions of the mouse *Jarid2* gene in BM and stromal vascular fat macrophages from VD(−) and VD( + ) HSC recipients, and these findings were confirmed by pyrosequencing (Supplementary Table 2). We identified an increase in the methylation status of several CpG sites in intron one within or near putative *Jarid2* enhancers between its two promoters in the BM from VD(−) HSC recipients. Methylation of these sites was preserved in SVF macrophages from 6-month-old VD(−) HSC recipients (Fig. 3G).

### Jarid2/PCG1α programming induces insulin resistance

**Fig. 4:**
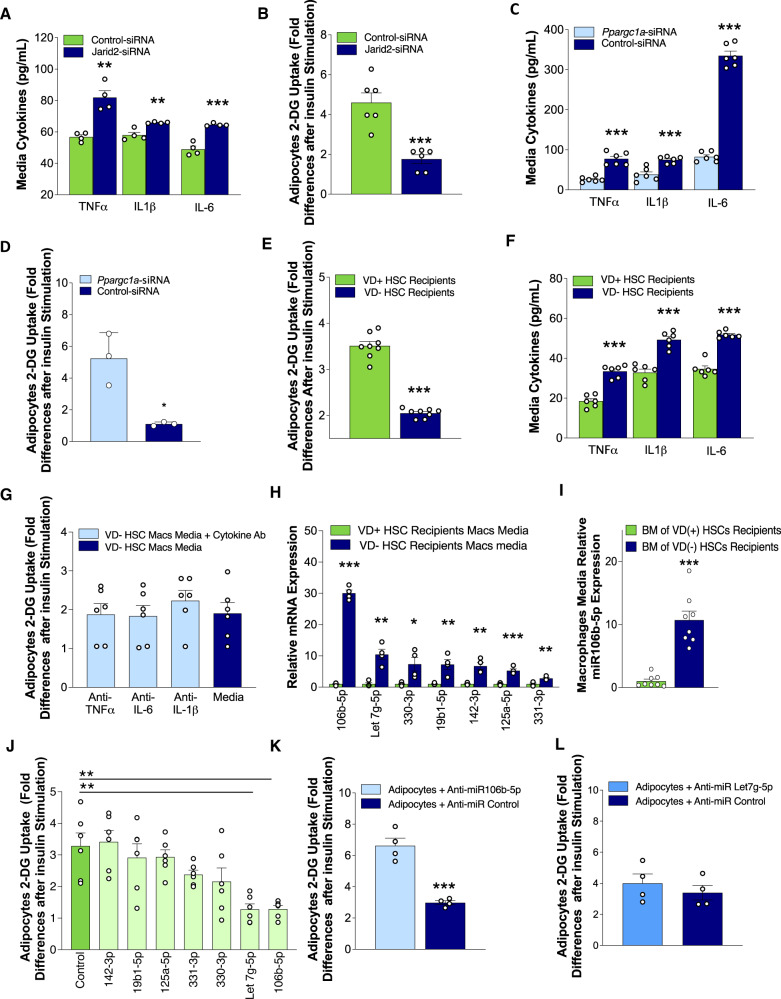
Activation of the Jarid2/Mef2/PGC1*α* immune cell program by in utero VD deficiency promotes pro-inflammatory cytokine and miRNA release. **A** Secreted cytokine levels in media (*n* = 4/group) in Jarid2-siRNA vs. control-siRNA-transfected peritoneal macrophages from VD( + ) FL-HSCs recipients. **B** Insulin-stimulated 2-DG uptake in 3T3-L1 adipocytes co-cultured with peritoneal macrophages from VD( + ) FL-HSCs recipients transfected with Jarid2-siRNA vs. control-siRNA (*n* = 6/group). **C** Secreted cytokine levels in media (*n* = 6/group) from *Ppargc1a*-siRNA vs. control-siRNA-transfected peritoneal macrophages from VD(−) FL-HSCs recipients**. D** Insulin-stimulated 2-DG uptake in 3T3-L1 adipocytes co-cultured with peritoneal macrophages from VD(−) FL-HSCs recipients transfected with *Ppargc1a*-siRNA vs. control-siRNA (*n* = 3/group). **E** Insulin-stimulated 2-DG uptake in 3T3-L1 adipocytes co-cultured with eWAT SVF macrophages from VD(−) or VD( + ) HSC recipients (*n* = 8/group). **F** Secreted cytokine levels in media (*n* = 6/group) from SVF macrophages from VD(−) or VD( + ) HSC recipients. **G** Insulin-stimulated 2-DG uptake in 3T3-L1 adipocytes co-cultured with SVF macrophages from VD(-) HSC recipients treated with or without cytokine-neutralizing antibodies (*n* = 6/group). **H** Relative miRNA content in exosomes secreted by peritoneal macrophages from VD(−) vs. VD( + ) FL-HSCs recipients (*n* = 8/group). **I** Relative miRNA content in exosomes secreted by peritoneal macrophages isolated from secondary recipients from the bone marrow of VD(+) primary recipients transplanted with VD(−) HSCs (*n* = 8/group). **J** Insulin-stimulated 2-DG uptake in 3T3-L1 adipocytes after transfection with miR mimics (*n* = 6/group). **K**, **L** Insulin-stimulated 2-DG uptake in 3T3-L1 adipocytes transfected with **K** miR-106b or **L** let7g-5p antagomir or control and cultured in conditioned macrophage media from VD(−) HSC recipients (*n* = 4/group). Data presented as mean ± SEM. *P < 0.05; **P < 0.01; ***P < 0.001 by two-tailed unpaired *t* test except in (**G**) and (**I**) where *P < 0.05; **P < 0.01; ***P < 0.001 by one-way ANOVA followed by Tukey’s multiple comparison test. Actual *P* values are shown in the source data file.

To test whether the macrophage epigenetic program activated in VD(−) HSCs induces adipose IR, we silenced *Jarid2* expression in peritoneal macrophages from VD( + ) HSC recipients. Silencing of macrophage *Jarid2* augmented inflammatory cytokine secretion (TNF*α*, IL-1β, IL-6) and induced adipose IR in co-culture experiments with 3T3-L1 adipocytes (Fig. 4A, B). Conversely, PGC1*α* deletion in peritoneal macrophages from VD(−) HSC recipients suppressed inflammatory cytokine release and improved adipose sensitivity in co-culture experiments with 3T3-L1 adipocytes (Fig. 4C, D), suggesting that activation of this inflammatory pathway in macrophages by in utero VD deficiency may be responsible for adipose IR.

To investigate how eWAT adipose tissue macrophages (ATM) from VD(−) HSC recipients induce adipose IR, we isolated SVF ATMs from VD(−) and VD( + ) HSC recipients and co-cultured these macrophages with 3T3-L1 adipocytes in transwell chambers. Adipocytes co-cultured with VD(−) HSC-recipient ATM had lower insulin-stimulated 2-DG uptake compared to those cultured with ATM from VD( + ) HSC recipients (Fig. 4E). Interestingly, ATM from VD(−) HSC recipients also demonstrated increased secretion of TNF*α*, IL-1β, and IL-6 without additional pro-inflammatory stimuli (Fig. 4F). However, the addition of cytokine-neutralizing antibodies did not improve the adipocyte IR in co-culture experiments with 3T3-L1 adipocytes (Fig. 4G), suggesting that the IR phenotype is not cytokine-driven. Therefore, analysis of cytokine levels was not pursued at later time points during the transplantation studies because the IR phenotype was cytokine-independent.

### Macrophage miR-106b-5p secretion causes adipocyte insulin resistance

Recently, microRNAs (miRNAs) have been shown to regulate chronic inflammation and IR^37,38^. Numerous immature miRNAs were downregulated within BM cells from VD(−) HSC recipients (Supplementary Table 3). However, the corresponding VD(−) HSC-recipient eWAT ATM media had increased levels of mature miRNAs, suggesting enhanced macrophage miRNA maturation and secretion. Similar to our previous finding in mice with macrophage-specific VDR deletion^33^, the most highly secreted miRNA from VD(−) HSC-recipient ATM macrophages was miR-106b-5p (Fig. 4H). Increased macrophage miR-106b-5p secretion persisted in secondary recipients from the bone marrow of VD(+) primary recipients transplanted with VD(−) HSCs (Fig. 4I). Transfection of mouse 3T3-L1 adipocytes with miRNA mimics of the most abundant secreted macrophage miRNAs identified in VD(−) recipient ATMs showed that miR-106b-5p and Let-7g-5p induced the most significant adipocyte IR (Fig. 4J). However, adipocytes exposed to conditioned media of ATMs from VD(−) HSC recipients and transfected with antagomirs of miR-106b-5p but not with Let-7g-5p demonstrated improved insulin sensitivity (Fig. 4K, L).

**Fig. 5:**
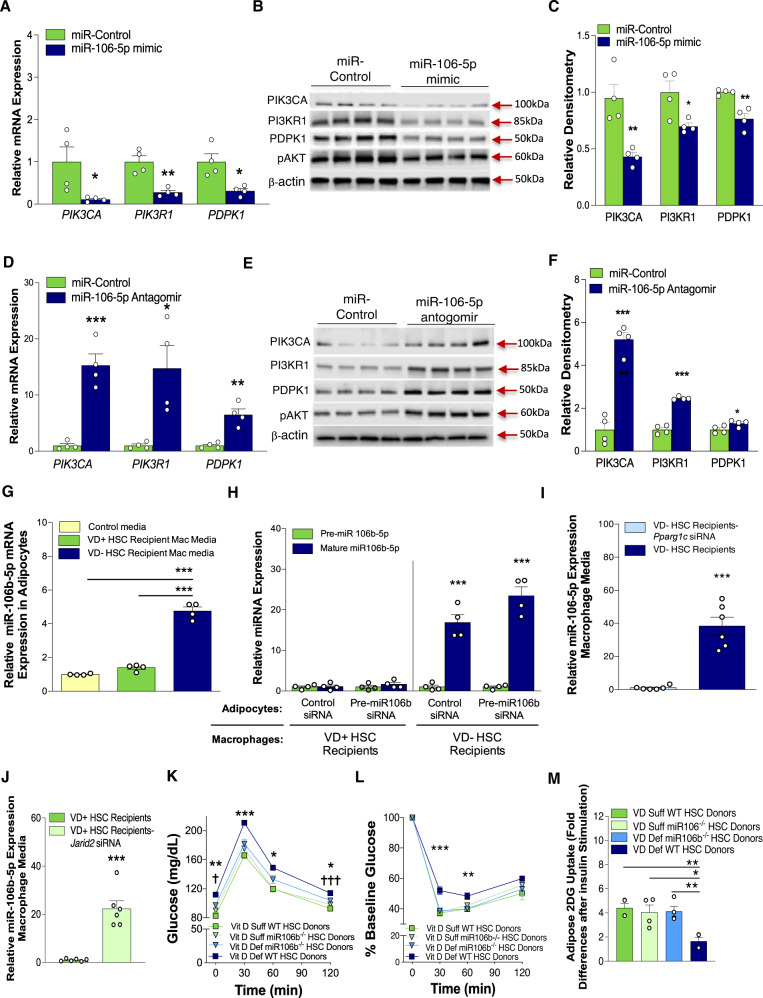
Macrophage miR-106b-5p mediates in utero VD deficiency-induced adipocyte IR. **A**–**C** Quantitative RT-PCR, Western blot analysis, and densitometry (normalized to β-actin protein levels) of the insulin signaling pathway in 3T3-L1 cells after transfection with miR-106b-5p mimic vs. control mimic (*n* = 4/group). **D**–**F** Quantitative RT-PCR, western blot analysis, and densitometry (normalized to β-actin protein levels) of the insulin signaling pathway in 3T3-L1 cells cultured in conditioned media from VD(−) HSC-recipient macrophages after transfection with anti-miR-106b or control (*n* = 4/group) from two independent experiments. **G** miR-106b-5p expression in adipocytes cultured in conditioned media from macrophages isolated from VD(−) or VD( + ) HSC recipients (*n* = 4/group). **H** Pre- and mature miR-106b-5p abundance in 3T3-L1 adipocytes transfected with pre-miR-106b siRNA vs. control-siRNA then cultured in conditioned media from macrophages isolated from VD(−) or VD( + ) HSC recipients (*n* = 4/group). Peritoneal macrophage media miR-106b-5p expression from **I** VD(−) HSC-recipient macrophages with or without *Ppargc1a*-siRNA, and **J** VD( + ) HSC-recipient macrophages with or without Jarid2-siRNA (*n* = 6/group). **K**–**M** Fetal HSCs from WT or miR-106b^−/−^ animals under VD(−) or VD(+) conditions were transplanted into VD( + ) WT recipients. **K** Glucose tolerance tests and **L** insulin tolerance tests (*n* = 8/group). **M** Insulin-stimulated 2-DG uptake in 3T3-L1 adipocytes co-cultured with peritoneal macrophages from WT or miR-106b^−/−^ animals transplanted with VD(−) or VD(+) HSCs (*n* = 6/group). Data presented as mean ± SEM. **A**, **C**, **D**, **F**, **H**, **I**, **J** *P < 0.05; ***P < 0.001 by two-tailed unpaired *t* test. **G**, **M** *P < 0.05; **P < 0.01; ***P < 0.001 by one-way ANOVA followed by Tukey’s multiple comparison test. **K**, **L** *P < 0.05; **P < 0.01; ***P < 0.001 VD(−) WT vs. all and ^†^*P* < 0.05; ^†††^*P* < 0.001 for VD( + ) WT vs. VD(−) miR-106b^−/−^. Actual *P* values shown in source data file.

To determine the mechanism by which miR-106b-5p promotes adipose IR, we utilized the computational prediction tool, TargetScan, to evaluate conserved putative miR-106b-5p targets within the insulin signaling genes and found that the phosphoinositide-3-kinase regulatory subunit 1 (PIK3R1) gene possesses miR-106-5p family binding sites in the human and mouse gene 3’UTRs. PIK3R1 encodes the regulatory subunits (p85α, p50α, and p55α). These subunits facilitate the phosphorylation of phosphatidylinositol bisphosphate (PtdIns 4,5)P2 by the catalytic subunits (p110α, p100β, p100δ), resulting in the generation of PtdInP3. This vital lipid messenger facilitates the membrane recruitment of the downstream kinases, 3-phosphoinositide dependent protein kinase 1 (PDPK1) and AKT to activate the insulin signaling pathway. To determine in our model if miR-106b-5p alters the expression of PIK3 subunits in adipocytes, we transfected 3T3-L1 adipocytes with a miR-106b-5p mimic. We found reduced transcript levels of the regulatory subunit p85α (PIK3R1), the catalytic subunit alpha (PIK3CA), and the downstream kinase PDPK1, which is responsible for AKT activation^39^. Notably, transcript levels of PIK3CB were not altered by miR-106b-5p mimic transfection. Western blot analysis confirmed decreased PIK3CA, PIKR1, and PDPK1 expression as well as decreased AKT phosphorylation (Fig. 5A–C). In contrast, transfection of 3T3-L1 adipocytes exposed to conditioned macrophage media from VD(−) HSC recipients with miR-106b-5p antagomir prevented the suppression of PIK3CA, PIK3R1, and PDPK1 expression, as well as AKT phosphorylation (Fig. 5D–F). These results suggest that miR-106b-5p induced-downregulation of the PIK3CA/PIK3R1/PDPK1/AKT signaling pathway is a primary mechanism driving adipose IR.

Adipocytes expressed little miR-106b-5p at baseline. However, adipocyte exposure to conditioned macrophage media from VD(−) HSC recipients increased mature miR-106b-5p abundance by sevenfold compared to VD( + ) HSC-recipient conditioned macrophage media (Fig. 5G). To define the cell responsible for producing mature miR-106b-5p, adipocytes were transfected with a pre-miR-106b siRNA to inhibit endogenous adipocyte miR-106b production and co-cultured with macrophages from VD(−) and VD( + ) HSC recipients. Increased abundance of mature miR-106b-5p in the media and exacerbation of IR in adipocytes was noted despite pre-miR-106b siRNA transfection when adipocytes were exposed to media from VD(–) HSC recipients, confirming that macrophages are the source of the miR-106b-5p that drives the IR phenotype (Fig. 5H and Supplementary Fig. 10). Finally, we found that silencing *Ppargc1a* in VD(−) HSC-recipient macrophages suppressed miR-106b-5p secretion (Fig. 5I) while silencing *Jarid2* in VD( + ) HSC-recipient macrophages promoted miR-106b-5p secretion (Fig. 5J). Overall, these results indicate that the Jarid2/Mef2/PGC1*α* immune cell program induced by VD deficiency in utero regulates macrophage miR-106b-5p secretion.

To confirm the role of macrophage miR-106b-5p in the IR phenotype, we transplanted VD(−) or VD(+) HSCs from miR-106^−/−^ or miR-106^+/+^ mice into VD-sufficient control mice. Recipients of VD(−) miR-106^−/−^ HSCs had improved glucose tolerance by GTT and improved adipose insulin sensitivity by ITT compared to VD(−) miR-106^+/+^ HSC recipients (Fig. 5K, L). These findings were supported by co-culture experiments with 3T3-L1 cells and macrophages from VD(−) or VD(+), miR-106^−/−^ or miR-106^+/+^ HSCs recipients, which showed that the absence of miR-106b-5p improved 3T3-L1 IR when cultured with macrophages from VD(−) HSC recipients (Fig. 5M), indicating that macrophage secretion of miR-106b-5p is critical for the adipose IR phenotype induced by in utero VD deficiency.

### Cord blood monocytes from vitamin D-deficient subjects cause adipocyte IR

**Fig. 6:**
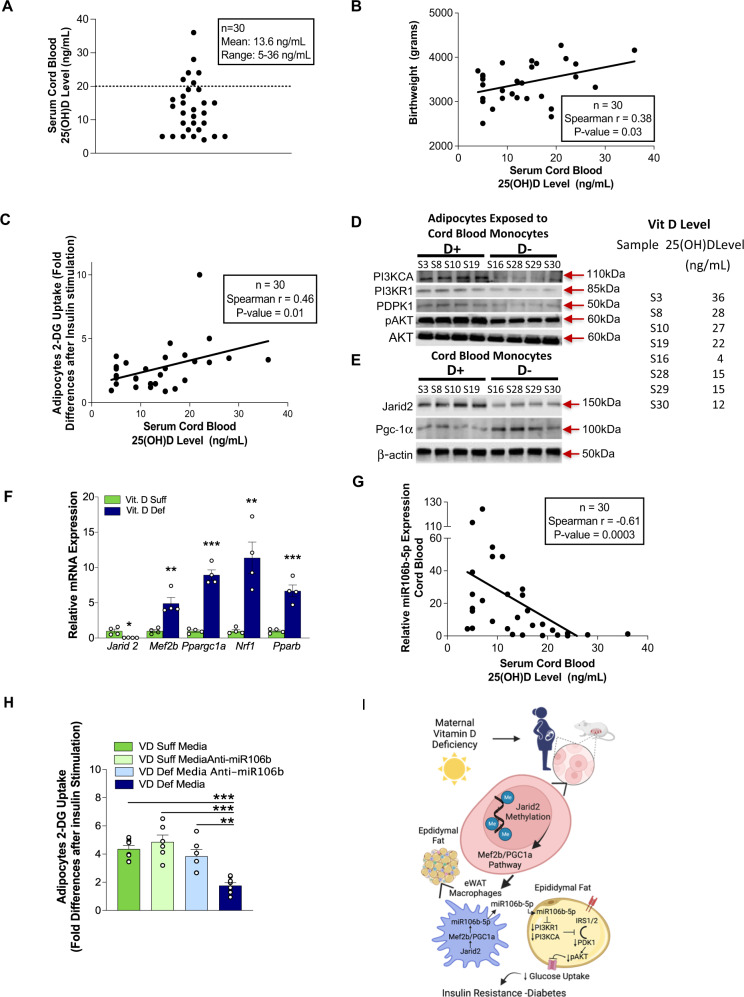
VD-deficient cord blood monocytes induce adipocyte IR. **A** Cord blood serum 25(OH)D levels from 30 healthy pregnant women at delivery. Mean and 95% confidence interval. **B** Correlation between cord blood serum 25(OH)D levels and birth weight using Spearman’s correlation coefficient. **C** Correlation between cord blood serum 25(OH)D levels and change in insulin-stimulated 2-DG uptake in 3T3-L1 adipocytes cultured in conditioned media of cord blood monocytes using Spearman’s correlation coefficient. Western blot analysis of insulin signaling pathway from 3T3-L1 adipocytes exposed to cord blood monocytes (**D**) and from Jarid2/Mef2/PGC1α network-related proteins of cord blood monocytes (**E**) (*n* = 4/group from two independent experiments). **F** Quantitative RT-PCR of mRNA expression of Jarid2/Mef2/PGC1α network-related genes from cord blood monocytes stratified by 25(OH)D level (25(OH)D < 20 ng/mL and ≥20 ng/mL) (*n* = 4/group)**. G** Correlation between cord blood serum 25(OH)D level and serum miR-106b-5p expression (*n* = 30) using Spearman’s correlation coefficient. **H** Insulin-stimulated 2-DG uptake in 3T3-L1 adipocytes transfected with miR-106b-5p antagomir and cultured in conditioned media from blood monocytes with 25(OH)D < 20 ng/mL or ≥20 ng/mL (*n* = 6/group)**. I** Mechanistic schematic diagram Created with BioRender.com. Data presented as mean ± SEM. **F** *P < 0.05; **P < 0.01; ***P < 0.001 by two-tailed unpaired *t* test. **H** **P < 0.01; ***P < 0.001 by one-way ANOVA followed by Tukey’s multiple comparison test. Actual *P* values are shown in the source data file.

To determine if VD deficiency during pregnancy in humans induces similar HSC cell reprogramming, we analyzed 30 healthy pregnant women prior to delivery and their full-term infants (Supplementary Table 4). Two-thirds of newborns were VD-deficient [25(OH)D ≤ 20 ng/mL], and cord blood 25(OH)D levels directly correlated with newborn birth weight (Fig. 6A, B), consistent with previous studies^40^. We found a direct correlation between cord blood 25(OH)D levels and 3T3-L1 adipocyte insulin sensitivity after exposure to conditioned media from cord blood monocytes (Fig. 6C). Adipocytes exposed to cord blood monocyte conditioned media from vitamin D-deficient mothers had lower PIK3CA, PIK3R1, and PDPK1 protein levels, as well as reduced pAKT, indicating that VD(−) monocyte/macrophage-induced dysregulation of the PIK3 pathway in adipocytes is conserved in humans (Fig. 6D). Moreover, cord blood monocytes from VD-deficient mothers had lower *Jarid2* expression and protein levels. *Mef2/PGC1a* expression and protein levels were also increased, resembling the epigenetic program in mouse immune cells exposed to VD deficiency in utero (Fig. 6E, F). Cord blood 25(OH)D levels also correlated inversely with cord blood plasma miR-106b-5p levels **(**Fig. 6G). Transfection of 3T3-L1 adipocytes with miR-106b-5p antagomir improved the insulin resistance induced by conditioned media from VD-deficient monocytes (Fig. 6H). These findings highlight the persistent effects of maternal vitamin D deficiency on offspring macrophage function to cause insulin resistance (Fig. 6I).

## Discussion

Despite recognizing the importance of environmental conditions in utero as contributing to adult disease, there is little data identifying which conditions during embryogenesis and which target tissues carry the epigenetic program that increases the susceptibility to IR in the offspring later in life. Vitamin D deficiency and insufficiency are highly prevalent at the time of delivery and concordant in both mothers and their neonates^40^. This study provides direct evidence that vitamin D deficiency in utero is sufficient to induce stable epigenetic programming in HSCs that can be transplanted to generate IR in vitamin D-sufficient recipient mice and is not reversed with postnatal vitamin D supplementation. This is the first demonstration that an epigenetic modification of a single, non-metabolically active tissue compartment is sufficient to induce type 2 diabetes. In our model, VD deficiency epigenetically suppresses *Jarid2* expression and activates the *Mef2*/*PGC1a* pathway in fetal HSCs. This program persists in recipient monocytes/macrophages, promoting adipose macrophage infiltration and miR-106b-5p secretion to suppress adipose PIK3 regulatory and catalytical subunits and AKT activity, causing IR. Lack of miR-106b-5p prevents the VD(−) HSCs from adoptively transferring IR to recipients. These data strongly suggest that IR is caused by epigenetic reprogramming of myeloid cells induced by vitamin D deficiency in utero, leading to activation of the *Jarid2/Mef2/PGC1α*/*miR-106b-5p* pathway both in humans and mice.

Epigenetic memory is defined as the stable propagation of gene expression induced by an environmental or developmental stimulus and may be categorized as cellular, transgenerational, or transcriptional^41^. Cellular memory refers to mitotically heritable transcriptional states and is elegantly illustrated in our model, in which an epigenetic program is induced by in utero VD deficiency in HSCs and then transmitted to the bone marrow and monocyte/macrophage lineages. Furthermore, the stability of this memory is highlighted by the secondary BM transplant experiments in which BM from VD-deficient HSC recipients was capable of inducing IR in VD-sufficient mice. Cellular memory requires Trithorax and PcG group proteins such as PRC2 to ensure stable transmission of these chromatin signatures through mitotic division^41–43^. However, the role of PRC2 in immune cells as a regulator of metabolic disease has not been previously described. In our study, we found that vitamin D deficiency in utero persistently downregulates *Jarid2* expression and activates the immune cell *Mef2/PGC1α* pathway in donor HSCs, recipient BM, and adipose macrophages despite postnatal VD supplementation, suggesting a stable epigenetic program in immune cells. DNA methylation is one crucial epigenetic mechanism to suppress essential genes during differentiation^44^. Evidence linking maternal VD status to global DNA methylation in offspring liver, adipose tissue, and leukocytes has been described in multiple animal models and pregnant women^25,45–48^. In this study, NGS confirmed that vitamin D deficiency promotes methylation of numerous CpG islands in putative enhancers of intron one between the two promoters of the mouse *Jarid2* gene in recipient BM, and importantly this methylation is conserved at six months in recipient adipose macrophages. Thus, VD-dependent regulation of *Jarid2* and, consequently, PRC2 may have the capacity to induce profound changes in the methylome by attenuating the function of a significant regulator of chromatin structure and function. Future studies using site-directed mutagenesis will be critical to confirm the methylated enhancer sites that influence *Jarid2* expression.

PGC1α is a central transcriptional regulator of mitochondrial biogenesis and function and is activated in pro-inflammatory adipose macrophages in the context of insulin resistance induced by a high-fat diet^49^. This study demonstrated that HSCs from vitamin D-deficient fetal donors consistently maintained *Mef2/PGC1α* pathway activation when transplanted into recipients. Moreover, the activated PGC1α network in recipient adipose tissue macrophages promoted a pro-inflammatory phenotype with increased secretion of miR-106b-5p, causing adipose IR. Previous studies indicate that overexpression of PGC1α increases exosome biogenesis and expression of the endosomal sorting complex required for transport (ESCRT), suggesting a role of mitochondrial bioenergetics in macrophage handling of miRNA cargo into exosomes for secretion^50^. Our previous data demonstrate that selective knockout of the macrophage VDR during embryogenesis promotes miR-106b-5p secretion that activates juxtaglomerular cells to induce hyperreninemic hypertension^33^. Together, these data identify that the Jarid2/Mef2/PGC1α program induced by vitamin D deficiency in utero enables the communication between innate immune cells, adipocytes, and JG cells to cause cardiometabolic disease.

The role of circulating miRNAs as signaling mediators of cell-to-cell communication has been identified in physiological and pathological conditions^51^. Multiple miRNAs have been linked directly to the regulation of chronic inflammation and insulin secretion in metabolic tissues in diabetic patients and mouse models of insulin resistance^37,38^. However, it is notable that miRNAs associated with insulin signaling genes are poorly conserved across species, perhaps implying that those that are conserved may be essential for insulin action and more clinically relevant^52–54^. Recent studies show that miR-106b is increased in human circulation in the setting of IR and is highly expressed in the skeletal muscle of both humans and mice^38,55,56^. Moreover, in skeletal muscle cells, overexpression of miR-106b impairs insulin-stimulated glucose uptake and GLUT4 transportation, suggesting an essential role of circulating and tissue-specific miR-106b in IR^57^. However, the cell or tissue type that secretes this miR-106b and the conditions that trigger it have not been previously identified. In this study, we provide evidence via multiple mouse models that macrophages epigenetically programmed in utero by vitamin D deficiency are a source of miR-106b-5p, which induces adipocyte IR.

Phosphoinositide-3-kinase (PIK3) is activated in response to insulin treatment and plays a critical role in mediating metabolic responses^58^. The PIK3 complex required for insulin action consists of the p110α (PIK3CA) catalytic subunit bound to one of several regulatory subunits (p85α, p55α, and p50α) encoded by PIK3R1 gene. In adipocytes, the PIK3CA catalytic subunit is the primary insulin-responsive PIK3 subunit^59^. Mice lacking adipose PIK3CA or the downstream kinase PDK1 exhibited glucose intolerance and liver steatosis^39,60^. Paradoxically in mice, homozygous or heterozygous deletion of the regulatory subunits p85α, p50α or p55α have enhanced insulin sensitivity^61–63^. The discrepancy in IR phenotype observed between these genetic models could be explained by the reduction of the regulatory subunits competing with the p110a/p85a complex for binding to insulin receptor substrate 1, enabling insulin-induced AKT activation^64^. Therefore, the balance between the p110α/p85α complex and free p85α might define the state of insulin sensitivity^65,66^. In this study, we found that adipose macrophages from VD(−) HSC recipients and vitamin D-deficient cord blood monocytes secrete miR-106b-5p, which enters into adipocytes and downregulates both the catalytic and regulatory subunits, thereby preventing insulin-stimulated AKT activation and adipose glucose uptake. It is possible that in our model, the increased miR-106b-5p shifts the balance towards greater suppression of p110α over p85α, thus leading to insulin resistance. The mechanism for p110α suppression in this model is unclear as p110α is not a direct miR-106b-5p target. Interestingly, other mouse models have demonstrated coupled decreases in p110α and p85α when one of the units is suppressed^66^. Further studies are required to identify the mechanism by which miR-106b-5p downregulates p110α. Overall, we delineate an unprecedented mechanism by which the miRNA secretome of VD(−) HSC-derived macrophages can modulate adipose insulin signaling to cause IR.

In summary, our findings provide evidence that an epigenetic immune program in response to in utero vitamin D deficiency is sufficient to cause IR by a miRNA-specific mechanism that enables communication from innate immune cells to adipocytes. This program activates the *Jarid2/Mef2/PGC1α* pathway in immune cells, which persists across both differentiation and transplantation, highlighting the durability of these changes in the offspring regardless of subsequent vitamin D status. Similar alterations in the *Jarid2/Mef2/PGC1α* pathway are replicated in cord blood monocytes from vitamin D-deficient mothers. These results identify the need for clinical trials to prove that the widespread screening and treatment of vitamin D deficiency in pregnant women will reduce the long-term risk of cardiometabolic disease in their children and subsequent generations.

## Methods

### Animals

Mice were housed within a temperature-controlled room (21–22 °C) under a 12 h light/dark cycle and allowed free access to food and water.

### Animal models

Four different mouse transplant models were used: (1) C57BL/6 CD45.2^+^ donors into C57BL/6, CD45.1^+^ recipients; (2) LDL receptor knockout donors expressing GFP^+/−^ (GFP^+/−^LDLR^−/−^) into GFP^−/−^ LDLR^−/−^ recipients; (3) miR-106b^−/−^ C57BL6 CD45.2^+^ donors into C57BL/6 CD45.1^+^ recipients; and (4) *Vav1Cre*^*+/−*^ *or Vav1Cre*^*−/−*^ *Jarid2*^*fl/fl*^ mice. To generate fetal liver hematopoietic stem cell (FL-HSC) donors, we obtained C57BL/6 CD45.2^+^ (Jax/Lab 002014), GFP^+/−^ C57BL/6 (Jax/Lab 004353), LDLR^−/−^ (Jax/Lab 002207), miR-106b^−/−^ CD45.2^+^ (Jax/Lab 008460) mice, *Vav1Cre* CD45.2^+^ (Jax/Lab 00861) and *Jarid2*^*fl/fl*^ CD45.2^+^ (Jax/Lab 031141). We generated GFP^+/−^ LDLR^−/−^ mice by crossing GFP^+/−^ C57BL/6 with LDLR^−/−^ mice. LDLR^−/−^ were initially crossed with mice that constitutively express the green fluorescent protein (GFP^+/−^) as donors to facilitate the identification of GFP-positive embryos by UV lamp and assessment of donor bone marrow chimerism in GFP^−/−^ recipients. C57BL/6 CD45.2^+^ were used as donors, and C57BL/6 CD45.1^+^ as recipients to facilitate the determination of engraftment without the influence of GFP. There were no differences in insulin resistance phenotype regardless of the engraftment verification model. To generate vitamin D-deficient or -sufficient [VD(−) or VD( + )] FL-HSC donors, we transitioned the dam’s diet 4 weeks prior to pregnancy to either vitamin D-deficient (Harlan TD.87095) or sufficient (Harlan TD.96348) diet^67^. Females were mated with vitamin D-sufficient males to prevent the effects of vitamin D deficiency on male fertility. At gestational day 13.5, VD(−) or VD( + ) FL-HSCs from males and females were harvested for transplantation. A subgroup of pregnant dams was allowed to progress to term. Pups born both vitamin D-deficient and sufficient were weaned to vitamin D-deficient or sufficient diets for 8 weeks. Glucose and insulin tolerance test were performed, and peritoneal macrophages were obtained for RNA expression. All experiments included male and female animals. Mice were euthanized using cervical dislocation according to institutional guidelines. E13.5 embryos are not viable after c-section. Protocols were approved by the Washington University Institutional Animal Care and Use Committee (Protocol 21-0127) and complied with ethical regulations for laboratory animal studies.

### Primary FL-HSC transplants and secondary BM transplants

Fetal liver cells at embryonic day 13.5 include HSCs with a high proliferative capacity, increasing donor engraftment by 10-fold compared to BM stem cell donors^68^. For fetal liver transplantation, vitamin D-deficient or sufficient C57BL6 or LDLR^−/−^ pregnant mice were sacrificed at 13.5 days gestation, with the vaginal plug counted as day 0.5^69^. For the GFP model, positive embryos were selected by UV lamp before dissecting each fetal liver. Fetal livers were then rinsed in sterile saline, followed by trypsinization for 15 min at 37 °C. Fetal liver cells were resuspended in cold DMEM with 5% fetal bovine serum (FBS, Gibco #16000044), filtered through a 70‐µm filter (BD #352350), centrifuged at 125×*g* for 10 min, re-filtered through a 40‐µm filter, and centrifuged at 125*×g* for 5 min. The cells were then rinsed in 15 mL of cold phosphate‐buffered saline (PBS), pelleted, resuspended in 1 mL of PBS, counted using a hemocytometer and adjusted to 10^5^ cells per µL. Cells were genotyped by PCR for the Sry sex-determining region of chr Y to create mixed male and female pools for donation. Fetal liver cells, including HSCs, were injected intravenously within 8 h into 8-week-old vitamin D-sufficient recipient male and female mice following lethal irradiation with 10 Gy from a ^137^Cs gamma irradiator source. For bone marrow transplantation, BM cells were isolated from 24-week-post-primarily transplanted recipients of VD(−) or VD(+) HSCs by flushing the femurs and tibias with ice-cold PBS^28^. Total BM was washed, triturated using a 24-gauge needle (Benson Dickson), collected by centrifugation at 300 *g* for 4 min, and diluted with PBS. After lysis of erythrocytes using Red Blood Cells Lysis buffer (Roche#11814389001), cells were counted. Eight-week-old vitamin D-sufficient C57BL6 (CD45.1^+^) or GFP^−/−^ LDLR^−/−^ male and female recipient mice were lethally irradiated with 10 Gy from a ^137^Cs gamma irradiator source. Within 6 h after irradiation, recipient BM was reconstituted with ~5 × 10^6^ donor marrow cells via a single injection. Eight weeks after either type of transplantation and reconstitution, recipient engraftment was evaluated by flow cytometry quantification of the percentage of CD45.2 or GFP positivity in peripheral leukocytes of recipients, with only those animals >87% chimeric used in experiments^28,33^.

### Metabolic assessment

*Blood samples*: Fasting serum glucose, cholesterol, triglycerides, and FFA were measured after 6 h fasting using commercially available kits. For glucose and insulin tolerance tests, transplant recipient mice were evaluated at 8 weeks or 24 weeks post-transplant. Mice fasted for 6 h before peritoneal injection with 10% D-glucose (1 g/kg, Pfizer, # 00409-7517-16) or insulin (0.75 U/kg, Humulin R # U-100). For both studies, tail vein blood glucose was assayed using a glucometer at baseline and 30, 60, and 120 min after injection^70^. Plasma insulin was assessed at 30 min during GTT (by electrochemiluminescence immunoassay). Hyperinsulinemic-euglycemic clamps and in vivo 2-deoxyglucose uptake (2-DG) assays were performed five days after double-lumen catheters were placed. Animals fasted overnight, and glucose turnover was measured in the basal state and during the clamp at 12 weeks post-transplant in conscious mice, as previously described^28,70,71^. Immediately after euthanasia, hind limb muscles and perigonadal fat were harvested, washed with PBS, and placed in liquid nitrogen until pending analysis. Frozen tissue samples were ground, boiled, and centrifuged. Accumulated 2-DG in the supernatant was separated by ion exchange chromatography using a Dowex 1-X8 (100–200 mesh) anion exchange column. Data are expressed as µmol/100 grams of tissue/min [(2-DG x mean blood glucose)/area under the curve]. *For insulin-stimulated adipocyte 2-DG uptake*, differentiated 3T3-L1 adipocytes or primary adipocytes were co-cultured for 72 h with peritoneal or stromal vascular macrophages or exposed to described conditioned media. For co-culture, macrophages (0.3 × 10^6^) were placed on inserts in transwell plates with 3T3-L1 adipocytes or primary isolated perigonadal adipocytes in the bottom chamber. After macrophage co-culture, adipocytes were serum-starved for 3 h, washed, and incubated with or without insulin (10 nM) for 30 min, then incubated for 10 min with radioactive 2-DG (PerkinElmer NEC 720A250UC). Cells were washed in cold Krebs–Ringer phosphate HEPES (KRPH), and after lysis, [^14^C] was determined by scintillation counting to measure 2-DG uptake^72^. Cytochalasin B, a glucose transport inhibitor (50 µM), was used to correct for non-specific background uptake. Data is presented as a ratio of 2-DG uptake after insulin stimulation to that of non-insulin-stimulated cells. Immunoblots for phospho-AKT (Ser 473) (Cell Signaling #4058 dilution 1 µg/mL), AKT (Cell Signaling #112580 dilution 0.5 µg/mL), and β-Actin (Cell Signaling #8457 dilution 0.5 µg/mL) were performed in homogenized perigonadal adipose tissue from HSC recipients with or without insulin stimulation (Humulin R) 1 µg/mL for 5 min. *For skeletal muscle 2-DG uptake*, primarily transplanted mice were fasted for 6 h, then paired soleus and extensor digitorum longus (EDL) muscles from anesthetized mice were excised and incubated using a 2-step incubation protocol^71^. For all incubation steps, vials were continuously gassed with 95% O_2_/5% CO_2_ and shaken in a heated water bath, and one muscle from each mouse was incubated in solution supplemented with 100 μU/ml of insulin (Humulin R) while the contralateral muscle was incubated in solution without insulin (basal) followed by three incubation steps as previously described^71^. After incubation with 2-DG for 15 min, muscles were rapidly blotted on filter paper dampened with incubation medium, trimmed, freeze-clamped, and stored at −80 °C. Muscles were homogenized, and 2-DG uptake was determined by scintillation counting.

### Isolation of peritoneal and adipose tissue macrophages and primary adipocytes for macrophage phenotype characterization and adipose 2DG uptake

#### Peritoneal macrophages

Unstimulated macrophages were collected following peritoneal PBS injection and placed in 12-well transwell inserts (Costar polycarbonate filters, 3-µm pore size) for co-culture with adipocytes or in 12-well plates for 6–8 h for media collection as previously described^28,67^. *Stromal vascular fraction isolation*. Mice were perfused through the right ventricle with 25 mL of ice-cold PBS. Epididymal fat pads, were excised and minced in PBS with 0.5% BSA. Collagenase I (1 mg/ml, Millipore Sigma SCR 103) was added before incubation with shaking/rotating, and digestion was stopped with pre-warmed KHB (Millipore Sigma). The cell suspension was filtered through a 250-μm filter and spun at 500 × *g* × 5 min to separate floating adipocytes from the stromal vascular fraction (SVF) pellet. The SVF pellet was resuspended in FACS buffer and incubated with anti-CD14 magnetic microbeads (Miltenyi Biotec 130-050-201) and F4/80 (Miltenyi Biotec, 130-110-443) to isolate ATM, which were then placed in 12-well plates with transwell inserts for adipocyte culture or in collagen-coated plates with DMEM plus 10% exosome-depleted FBS for 6 h for media miRNA expression and cytokine analysis^73^.

#### Primary adipocytes

Perigonadal fat pads were initially treated with collagenase as described above, but then floating adipocytes were spun at 100 × *g* × 1  min. Buffer and SVF pellet underneath the floating adipocytes were removed, and cells were washed with pre-warmed KRPH. Adipocytes were transferred into collagen-I-coated 12-well plates (0.5 × 10^6^ cells/plate) and incubated at 37 °C for 60 min before starting the 2-DG protocol.

### 3T3-L1 adipocyte differentiation

Murine 3T3-L1 pre-adipocytes (American Type Culture Collection CL-173) were grown, maintained, and induced to differentiate using a standard protocol^74^. Fully differentiated adipocytes (12 days post differentiation induction) were maintained in DMEM supplemented with 10% FBS (Millipore Sigma) until two days before experimentation when cells were fed with 10% calf serum (Millipore Sigma). Prior to experiments, the media was changed to serum-starved DMEM (low glucose) for 2–3 h.

### Macrophage and adipocyte co-cultures

Transwell chambers were utilized (Costar polycarbonate filters, 3-µm pore size) as previously described^28^ for co-cultures. Membranes and 12-well plates were coated with fibronectin (Sigma Catalog # 341631) overnight at 4 °C. Peritoneal or SVF macrophages were cultured in DMEM plus 10% exosome-depleted FBS for 6 h, and media was tested for miRNA expression and cytokine levels prior to co-culture. Differentiated 3T3-L1 adipocytes were grown to 80–90% confluence in 12-well plates, with a maximum of 0.5 × 10^6^ cells/well. Macrophages (0.3 × 10^5^ cells/well) were added to the transwell upper chamber, with adipocytes in the lower chamber. Cells were co-cultured for 72 h in DMEM/F12 media with 10% exosome-depleted FBS with 2-DG quantification as described above. Some 3T3-L1 cells were cultured only with macrophage-conditioned media but additionally incubated with 0.2 µg/ml TNFα-, IL-1β-, or IL-6-neutralizing antibodies (R&D Biosystems MAB4101) for 72 h, with 2-DG quantification as described above^71^.

### Analysis of monocytes and adipose tissue macrophages (ATMs) and HSCs in the fetal liver by conventional flow cytometry

Monocyte and eWAT SVF macrophage cell surface marker analysis was performed using a FACStar Plus as previously described^75,76^. After isolation, including CD11b selection with microbeads (Catalog #130-049-601) cells were resuspended in flow cytometry buffer (BD Bioscience # 51-2091KZ), and >10^5^ cells were analyzed for each sample. Monocytes were incubated with 10 µg/ml of anti-mouse APC-CD45.1 (BioLegend, #110714) or 10 µg/ml of anti-mouse PE-CD45.2 (BioLegend #109808) for 15 min on ice, then washed before flow cytometry with utilization of 5 µg/ml APC mouse IgG2a/K (BioLegend #400219) and 5 µg/ml of PE mouse IgG2a/K (BioLegend #400211) isotype controls. EGFP-positive cells were excited at 488 nm and measured by flow cytometry at 530 nm. To determine the percentage of ATM cells, CD11b+ SVF from epididymal fat were incubated with 10 µg/ml of anti-mouse PE-F4/80 (BioLegend, #12-4801-82) with the utilization of 5 µg/ml PE rat IgG2a K isotype Control (BioLegend#12-4321-80). To further characterize ATM cells, CD11b+ SVF were stained with 4 µg/ml anti-CCR7 APC-eFluor780 (#47-0271-82 eBioscience) and 4 µg/ml anti-CD86 eFluor450 (#48-0862-80 eBioscience) for M1 macrophage marker expression and 20 µg/ml anti-CD163-Cy5.5 (# M130 Bioss USA) and 4 µg/ml anti-CD206-Alexa700 (#FAB2535N R&D Systems) for M2 macrophage marker expression. Fetal liver cells were incubated with antibody cocktails for long-term hematopoietic stem cell (HSC) antibodies: 10 µg/ml Alexa Fluor® 700 or PE anti-mouse lineage cocktails (#79923 or #78035 BioLegend), 4 µg/ml Sca1- Ly-6A-Alexa Fluor® 700 (#56-5981-82 eBioscience), 4 µg/ml CD117-APC (#17-1172-81 eBioscience), 4 µg/ml CD150-APC 780, (#47-1502-82 eBioscience); Short-term HSCs antibodies: 4 µg/ml CD34 - PE-Cy7 (#25-0349-42 eBioscience), 5 µg/ml Flt3 - PE-Cy5 (#15-1351-82 eBioscience); 5 µg/ml pro B cell: B220-efluor450 (#48-0452-6B2 eBioscience), 4 µg/ml CD43-PE (#12-0431-82 eBioscience); pro T cells: 4 µg/ml CD25-PE (#12-0259-80 eBioscience), CD44-APC (#17-0441-82 eBioscience); 8 µg/ml CMP/GMP: CD16/32-eFluor450 (#48-0161-82 eBioscience); 5 µg/ml MDP/CDP: CD115 APC-eFluor780 (# 47-1152-82 eBioscience).

Cells were acquired using FACStar Plus flow cytometer. Cell aggregates, dead, and cellular debris were excluded based on FSC/SSC. Batch analysis by Flow Jo version 9.6.2 was used for gating consistency and selection of positive populations. Unstained samples were used to control background autofluorescence signals. Flow cytometry data are presented as the percentage of fluorophore- or GFP-positive live cells or as the ratio of M1/M2 percentage of fluorophore-positive live cells in detailed ATM characterization. The gating strategy of the HSC analysis is presented in Supplemental Fig. 2.

### Analysis of immune cell populations in tissues by spectral flow cytometry

Epidydimal adipose tissue (eWAT), inguinal adipose tissue (SubCu) and liver tissues were collected. Lymph nodes were excised from SubCu. Tissues were washed with PBS, minced to small pieces with scissors and digested. Adipose tissues were digested during 30 min at 37 °C in phenol red-free DMEM + 0.5% BSA + collagenase D (1 mg/mL; #11088882001, Roche). Upon digestion, cells were sieved through 100-μm cell strainers and spun down at 500 × *g*, 10 min, 4 °C without breaking. Supernatants were carefully aspirated and the pellet containing (SVF) was incubated with red blood cell lysis buffer (#A1049201, Gibco) buffer for 3 min. 10 ml of cold DMEM was added and cells were spun at 500 × *g*, 10 min, 4 °C. Cells were then resuspended in PBS and counted to obtain total SVF counts in both fat pads from each mouse. Liver samples were processed according to ref. ^77^. Briefly, minced tissue was digested in phenol red-free DMEM + 0.5% BSA + collagenase D (1 mg/mL; #11088882001 Roche) + DnaseI (5 mg/mL; #10104159001 Roche) for 30 min at 37 °C. Cells were then sieved through 70-µm cell strainers and centrifuged at 50×*g* for 3 min at 4 °C to initially separate hepatocytes (pellet) and non-parenchymal cells (NPCs) (supernatant). The supernatant containing NPCs was collected and NPC suspension was centrifuged at 163 *g* for 7 min at 4 °C to pellet the NPCs. NPC pellet was resuspended in red blood cell lysis buffer 1 mL and incubated for 5 min, then washed and centrifuged at 163×*g* for 7 min at 4 °C to re-pellet the NPCs. In total, 2–3 × 10^5^ cells were incubated with Live-or-Dye 665/685 viability dye (1000× diluted according to manufacturer’s instruction; #32013, Biotium) in PBS for 15 min at 4 °C, in 96-well V-bottom plate. Cells were washed and resuspended in FACS buffer (DPBS, 2% FBS, 2 mM EDTA). TruStain FcX PLUS (2.5 μg/mL; #156604 Biolegend) was added to block non-specific Fc receptor binding, and cells were incubated for 10 min. Antibody cocktail of 1 μg/ml CD45.2—BV750 (#747251 BD Biosiences), 1 μg/ml CD11b—BV605 (#101257 Biolegend), 1 μg/ml CD11c—PE-Cy5 (#117316 Biolegend), 1 μg/ml F4/80—PE-Cy7 (#123114 Biolegend), 1 μg/ml CD3—BV650 (#100229 Biolegend), 1 μg/ml CD4—PE-Dazzle594 (#100566 Biolegend), 1 μg/ml CD8—PE (#100708 Biolegend), 1 μg/ml CD19 -BV711 (#115555 Biolegend), 2 μg/ml NK1.1—BV480 (#746265 BD Biosciences), 0.5 μg/ml Ly-6C—BV570 (#128029 Biolegend), 2.5 μg/ml Ly-6G—FITC (#127606 Biolegend), 1 μg/ml Siglec-F—APC-Cy7 (#565527 BD Biosciences), 2.5 μg/ml FcεRIα—Alexa Fluor® 700 (#134324 Biolegend), 1 μg/ml CD45.1—BV421 (#110732 Biolegend) was added, and cells were incubated for 30 min at 4 °C. Cells were washed 3× with FACS buffer, resuspended in ice-cold PBS and acquired on Cytek Nothern Lights 3-laser spectral cytometer.

An unmixing of the spectral data was performed using SpectroFlo software (Cytek). For unbiased analysis, FCS files were processed using OMIQ analysis platform. Doublets, debris, and dead cells were excluded, and live CD45+ were downsampled to an equal number of cells from each mouse. Data from independent experiments were pooled (15,472 events per condition for eWAT, 8940 events per condition for SubCu, 4148 events per condition for liver), and uniform manifold approximation and projection (UMAP)^78^ was used to visualize the data. FlowSOM algorithm was used to generate filters, which were then manually inspected and adjusted in some cases. The total number of cells in identified filters were calculated from total SVF cell counts and CD45 percentages in each sample (mouse).

### F4/80 immunofluorescence staining

For tissue sections, ketamine/xylazine-anesthetized mice were perfused for 10 min with 4% paraformaldehyde before perigonadal fat pads were collected and immersed in the same fixative for 12–15 h at 4 °C. After rinsing and PBS wash, the tissue was dehydrated with gradual steps of EtOH and paraffin-embedded. Adipose tissue was cut into 3–4 μm sections, then slides were deparaffinized, rehydrated, and blocked for endogenous peroxidase activity (1% H_2_O_2_ in TBST). Following the manufacturer’s recommendations, the slides were stained with F4/80-specific antibodies (1:300 Abcam ab6640). F4/80-positive cells were counted in 15 fields with a ×20 objective. A similar protocol was used for F4/80 immunohistochemistry in 5-μm paraffin sections of brown adipose tissue and muscle, using a 1:100 dilution of the Abcam ab6640 antibody and hematoxylin counterstaining. Adipocyte size was measured from a minimum of 200 adipocytes per mouse using ImageJ software.

### Monocyte adhesion and migration

Briefly, 3 × 10^5^ CD14^+^eWAT SVF macrophages were added to fibronectin-coated plates to assess adhesion and incubated for 4 hr at 37 °C before adhered cells were stained crystal violet and absorbance was quantified. Transwell migration assays were performed (Costar polycarbonate filters, 5-μm pore size) as previously described.^79^ Membranes and 12-well plates were coated with fibronectin (5 μl/mL; Life Technologies) overnight at 4 °C. CD14^+^eWAT SVF macrophages (0.3 × 10^5^ cells/well) were added to the upper chamber, and MCP-1 (100 ng/well; Sigma) in 0.8% agarose solution was added to the lower chamber to stimulate migration. Cells migrating into the lower chamber after 8 h of incubation were manually counted and presented as a percentage of cells migrated.

### Microarray and bioinformatics analysis

Purified DNA-free RNA from primary recipients’ BM cells was quantified, and quality was assessed using a Nanodrop ND‐100 spectrophotometer and hybridized to Affymetrix GeneChip miRNA 4.0 microarrays by Washington University’s Genome Technology Access Center. The array signal data was processed with Partek Genomics Suite 6.6 (Partek, St Louis, MO). Upregulated genes (ratio >1.19 and nominal *P* < 0.05) and downregulated genes (ratio <0.94 and nominal *P* < 0.05) in BM of VD- FL-HSC recipients were used as input for enrichment pathway analysis using Enrichr, Embryonic Stem Cells Atlas of Pluripotency Evidence (ESCAPE), gene Ontology (GO), Kyoto Encyclopedia of Genes and Genomes (KEGG), and Wiki-pathway databases. RNA sequences from bone marrow array data (GSE158763) have been deposited in the NCBI GEO repository.

### Methylation analysis

Genomic DNA from bone marrow and 6-month-old eWAT ATM was obtained. Next-generation sequencing methylation analyses were performed by EpigenDx, Inc. (Hopkinton, Massachusetts, United States). 500 ng of extracted DNA samples were bisulfite-modified using the EZ-96 DNA Methylation-Direct KitTM (ZymoResearch; Irvine, CA; cat# D5023) per the manufacturer’s protocol. All bisulfite-modified DNA samples were amplified using separate multiplex or simplex PCRs. PCR products from the same sample were pooled, and libraries were prepared using a custom Library Preparation method created by EpigenDx. Library molecules were purified using Agencourt AMPure XP beads (Beckman Coulter; Brea, CA; cat# A63882). Barcoded samples were then pooled in an equimolar fashion before template preparation and enrichment were performed on the Ion ChefTM system using Ion 520TM & Ion 530TM ExT Chef reagents (Thermo Fisher; Waltham, MA; cat# A30670). Following this, enriched, template-positive library molecules were sequenced on the Ion S5TM sequencer using an Ion 530TM sequencing chip (cat# A27764). FASTQ files from the Ion Torrent S5 server were aligned to the local reference database using open-source Bismark Bisulfite Read Mapper with the Bowtie2 alignment algorithm. Methylation levels were calculated in Bismark by dividing the number of methylated reads by the total number of reads. The % methylation increase was calculated as CpG methylation of recipients of HSC VD(−) – CpG methylation of HSC VD(+) recipients/CpG methylation of HSC VD(+) recipients.

### Exosome isolation

Media was centrifuged at 2000*×g* for 30 min. The supernatant was transferred to a new tube and centrifuged at 100,000×*g* for 18 h. Density-gradient-based isolation was then performed using the Total Exosome Isolation kit (Invitrogen)^80^. The purity of exosome vesicles was confirmed by western blot showing the presence of EV marker protein expression as previously described^33,80^.

### MicroRNA and mRNA expression via RT-qPCR

miRNA purification and isolation from 3T3-L1 cells were performed using the *mir*Vana miRNA kit (Invitrogen Ambion) and anti-miR (AM10067). RTq-PCR was performed using the TaqMan reagent kit, with the relative expression of miRNA calculated by the comparative threshold cycle method relative to miR-39 from *C. elegans*. For media assays, we spiked in miR-39 as an exogenous housekeeping miRNA control prior to extraction (Qiagen #219610). TaqMan primers were obtained from Life Technologies for miRNAs 106b-5p (#000442), 106b-3p (#002380), let7g-5p (#002282), 142-3p (#000464), 330-3p(#001062), 19b-5p (#002425), 125a-5p (#002198), 331-3p (#000545), U6 (#4427975) and 39 (#467942). mRNA RTq-PCR was performed with the GeneAmp 7000 Sequence Detection System using the SYBR® Green reagent kit (Applied Biosystems)^33^. We used the following mouse oligonucleotides; for *Jarid2* forward 5′-GCGGTAAATGGGCTTCTTGG-3’; *Jarid2* reverse 5’- TGCTAGTAGAGGACACTTGGGA-3’; *Ppargc1a* forward 5′-AGCCTCTTTGCCCAGATCTT-3’; *Ppargc1a* reverse 5′-GGCAATCCGTCTTCATCCAC-3’, *Mef2b* forward 5′-GACCGTGTGCTGCTGAAGTA-3; *Mef2b* reverse 5′-AGCGT CTCGAGGATGTCAGT-3’; *Nrf1* Forward 5′-GTACAAGAGCATGATCCTGGA-3’; *Nrf1-*reverse 5′-GCTCTTCTGTGCGGACATC-3’; *Atp5g* forward 5’-AGTTGGTGTGGCTGGATCA-3’; *Mrpl32* forward, 5’-AAGCGAAACTGGCGGAAAC-3’; *Mrpl32* reverse, 5’-GATCTGGCCCTTGAACCTTCT-3’; *PDPK1* forward, 5’-CCACTGAGGAAGATCGACAGAC-3’; *PDPK1* reverse, TCTGCTCAGCTTCACCGCATTC-3’; *PIK3CA* forward, 5’-CACCTGAACAGACAAGTAGAGGC-3’; *PIK3CA* reverse, 5’-GCAAAGCATCCATGAAGTCTGGC-3’; *PIK3R1* forward, 5’-CAAACCACCCAAGCCCACTACT-3’; *PIK3R1* reverse, 5’- CCATCAGCAGTGTCTCGGAGTT-3’. All assays were done in triplicate, with data expressed as relative expression of mRNA normalized to mouse ribosomal protein *Mrpl32*.

### Plasmids and small interfering RNA transfection

3T3-L1 cells were transfected with mirVana miR-106b (LifeTechnology MC10067), let 7g-5p (LifeTechnology MC11758), 330-3p (LifeTechnology MC10732), 19b-5p (LifeTechnology MC13042), 142-3p (LifeTechnology M C10398),125a-5p (LifeTechnology MC12561), 331-3p (LifeTechnology MC10881) mimics or miR-106b or Let7g antagomirs (AM10067, MH11758 respectively). 72 h after transfection, 3T3-L1 cells were evaluated for 2-DG uptake and mRNA expression in lysates by RT-qPCR. 3T3-L1 cells were also transfected with pre-miR-106b siRNA sense oligonucleotides 5’-CCU AAU GAC CCU CAA GCC GUU-3 and antisense 5’-CGG CUU GAG GGU CAU UAG GUU-3’ then exposed to VD( + ) HSC or VD(-) HSC-recipient peritoneal macrophage media for 72 h before assessment of pre-miR-106-5p or mature miR-106b-5p. Peritoneal macrophages from VD( + ) HSC or VD(-) HSC recipients were transduced with lentivirus containing sense either Jarid2-siRNA (LifeTechnology 4390771), *Ppargc1a*-siRNA (LifeTechnology, AM16708), or control-siRNA (LifeTechnology AM461**)** for 48 h and mRNA expression was determined as previously described^81^.

### Western blot analysis

3T3-L1 adipocytes were homogenized in RIPA lysis buffer containing protease and phosphatase inhibitors. Lysates were clarified, centrifuged, and resolved by SDS-PAGE. Samples were transferred to PVDF membranes that were subsequently probed with the following antibodies for protein and phosphoprotein detection: 9 µg/ml anti-PIK3CA (ab40776, Abcam), 4 µg/ml anti-PI3KR1 (ab191606 Abcam), 3 µg/ml anti-PDPK1 (ab52893, Abcam,) 1:1000 dilution ant-AKT (9272 S, Cell Signaling Technology), 1:500 dilution anti-pAKT (9271L, Cell Signaling Technology) and 1:2000 dilution β-actin (3700S, Cell Signaling Technology). Protein levels were quantified using Image Studio Lite Ver 5.2 (LI-COR) and normalized to β-actin protein levels.

### Human samples

Specimens & data were obtained in a de-identified manner from the Women and Infant Specimen Consortium (WIHSC) ICTS/CTSA ULI PR000448 IRB#201013004 consisting of 30 women with singleton pregnancies resulting in full-term vaginal or C-section delivery that fulfill the inclusion criteria were recruited during the day 7 am to 4 pm at Barnes and Jewish hospital by the Women and Infant Specimen Consortium (WIHSC) from 06/26/2017 to 05/15/2018. Sex from singleton was specified in Supplementary Table 4. Racial identity was provided by the Women and Infant Specimen Consortium (WIHSC) based on skin color. Intrauterine growth retardation, small/large for gestational age, diabetes (gestational, type 1, and type 2 DM), preeclampsia, chorioamnionitis, acute infection (fever or active herpes), and moderate or severe alcohol or drug abuse during pregnancy were excluded. Venous cord blood plasma was collected for 25-hydroxyvitamin D levels (LC-MS/MS)^75,76^ and quantification of miRNA expression from plasma exosomes^80,82^. Cord blood monocytes were isolated as previously described^81^. Monocytes were stabilized for 2 h in 100% serum from the original patient to mimic in vivo conditions for experiments^75,76^. Isolated monocytes were co-cultured with 3T3-L1 adipocytes in transwell chambers for insulin-stimulated 2-DG studies. Monocyte mRNA was also assessed by qPCR^81^.

### Statistical analysis

Experiments were carried out in duplicate or triplicate, with “*n*” referring to the number of distinct samples. Gaussian distribution was verified by Kolmogorov–Smirnov distance. Parametric data are expressed as mean ± SEM and analyzed by two-sided *t* tests, paired or unpaired as appropriate, or by one-way ANOVA and Tukey’s post-test for more than two groups. Statistical analysis was carried out using GraphPad Prism version 8.4.3.

### Reporting summary

Further information on research design is available in the Nature Portfolio Reporting Summary linked to this article.

## Supplementary information

Supplementary Information

Reporting Summary

## Data Availability

RNA sequences from bone marrow array data (GSE158763) have been deposited in the NCBI GEO repository. All data relating to the findings within this manuscript can be found within the manuscript, figures, or supplementary information.  Source data are provided with this paper.

## Source data

Source Data
