# Supplementary material for: Embryonic vitamin D deficiency programs hematopoietic stem cells to induce type 2 diabetes
Source: Nat Commun. 2023 Jun 13;14:3278. doi: 10.1038/s41467-023-38849-z (PMC10264405; doi:10.1038/s41467-023-38849-z)
Supplement: Supplementary file 1 — Supplementary Information [file 41467_2023_38849_MOESM1_ESM.pdf]

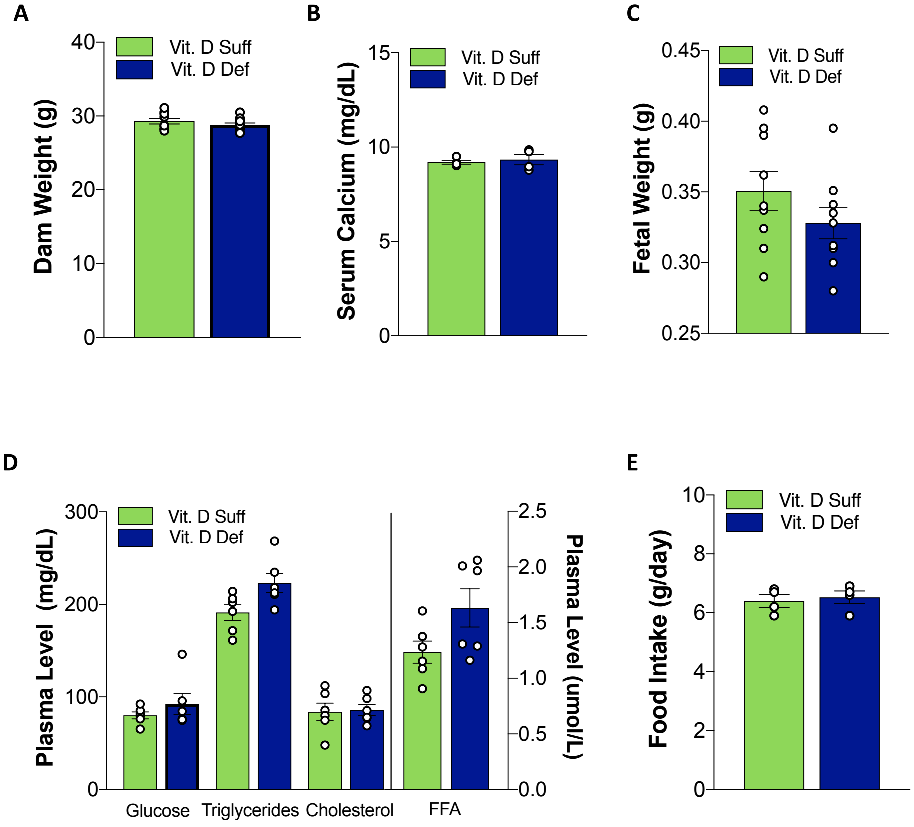

Supplemental Figure 1. Maternal vitamin D deficiency during pregnancy does not alter metabolic parameters in dams. (A) Dam weight (n=9 vitamin D sufficient, n=10 vitamin D deficient), (B) dam serum calcium (n=4 per group), (C) fetal weight (n=7 per group), (D) dam plasma fasting glucose and lipids (n=6 per group), and (E) dam food intake (n=4 per group) in vitamin D-sufficient or -deficient mice pregnant at 13 days and their fetuses. Data presented as mean ± SEM.

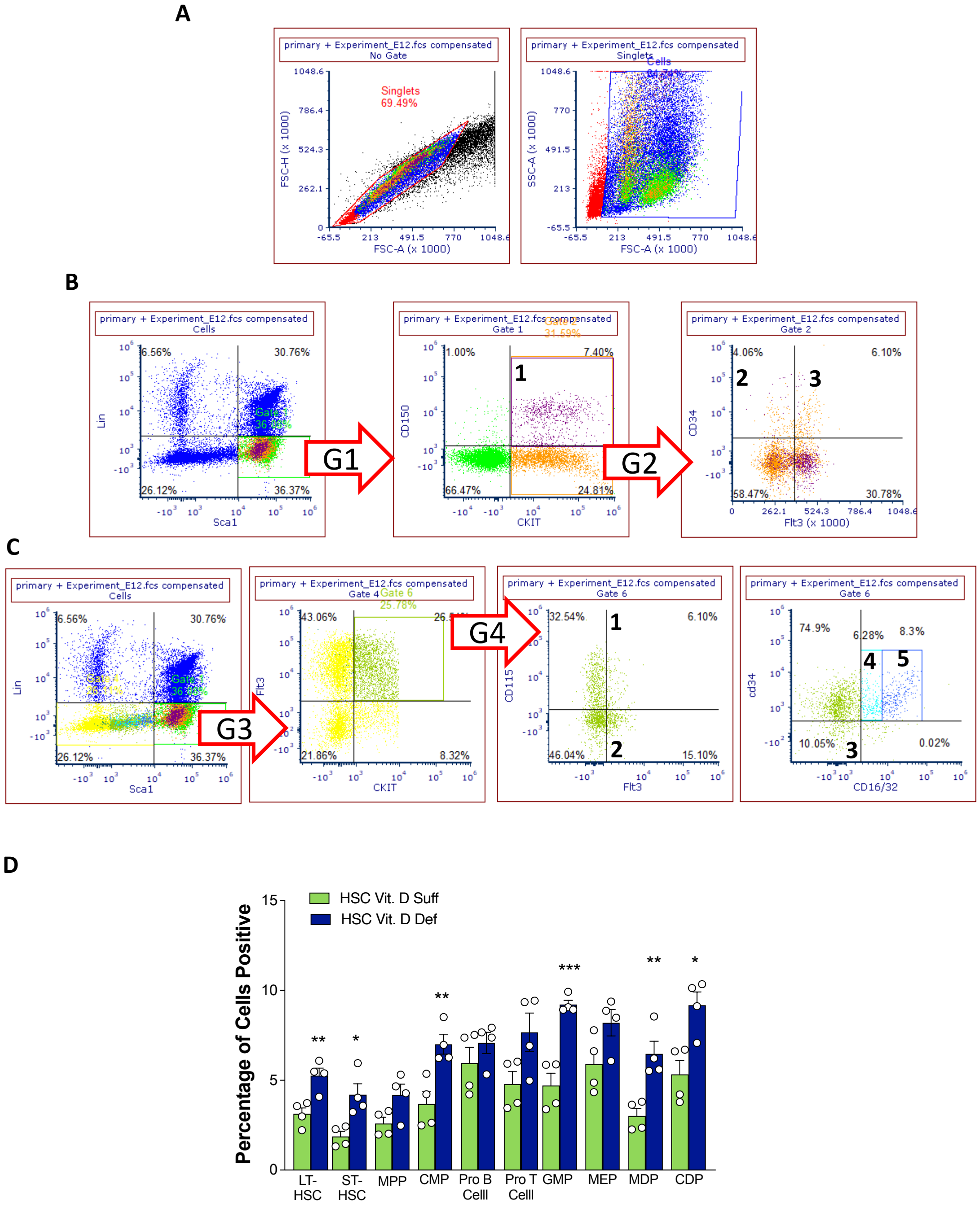

Supplemental Figure 2. The Prevalence of Myeloid Progenitors and Long-term and Short-term HSCs is Increased in Fetal Liver Donor Cells Isolated from VD deficient Embryos. Fetal liver cells were isolated from VD sufficient and deficient dams at day 13.5 and subjected to flow cytometry analysis to determine the prevalence of long-term (LT) and short-term (ST) HSCs, common myeloid progenitors (CMP), granulocyte-macrophage progenitors (GMP), megakaryocyte erythroid progenitors (MEP), and macrophage dendritic cell progenitors (MDP). (A-C) Gating strategy of HSC characterization. (A) Gating of singlets and cells based on FSC and SSC properties. (B) Gating strategy for identification of (# 1) long-term HSCs (LT-HSCs), (#2) short-term HSCs (ST-HSCs), and (#3) multipotent progenitors (MPP). (C) Gating strategy for identification of (# 1) macrophage dendritic cell progenitors (MDP) and (# 2) conventional dendritic cell progenitors (CDP). c-Kit$^+$ Flt3$^+$ were also plotted by CD16/32 CD34 identifying (#3) megakaryocytes erythroid progenitors (MEP); (# 4) common myeloid progenitors (CMP); and (# 5) granulocyte-macrophage progenitors (GMP). (D) Summary of the abundances of hematopoietic cell progenitors from VD- vs. VD+ FL-HSCs. (n= 4/group) Data presented as mean ± SEM. *p<0.05,**p<0.01, ***p<0.001 by two-tailed unpaired t-test. Actual p values are shown in the source data file.

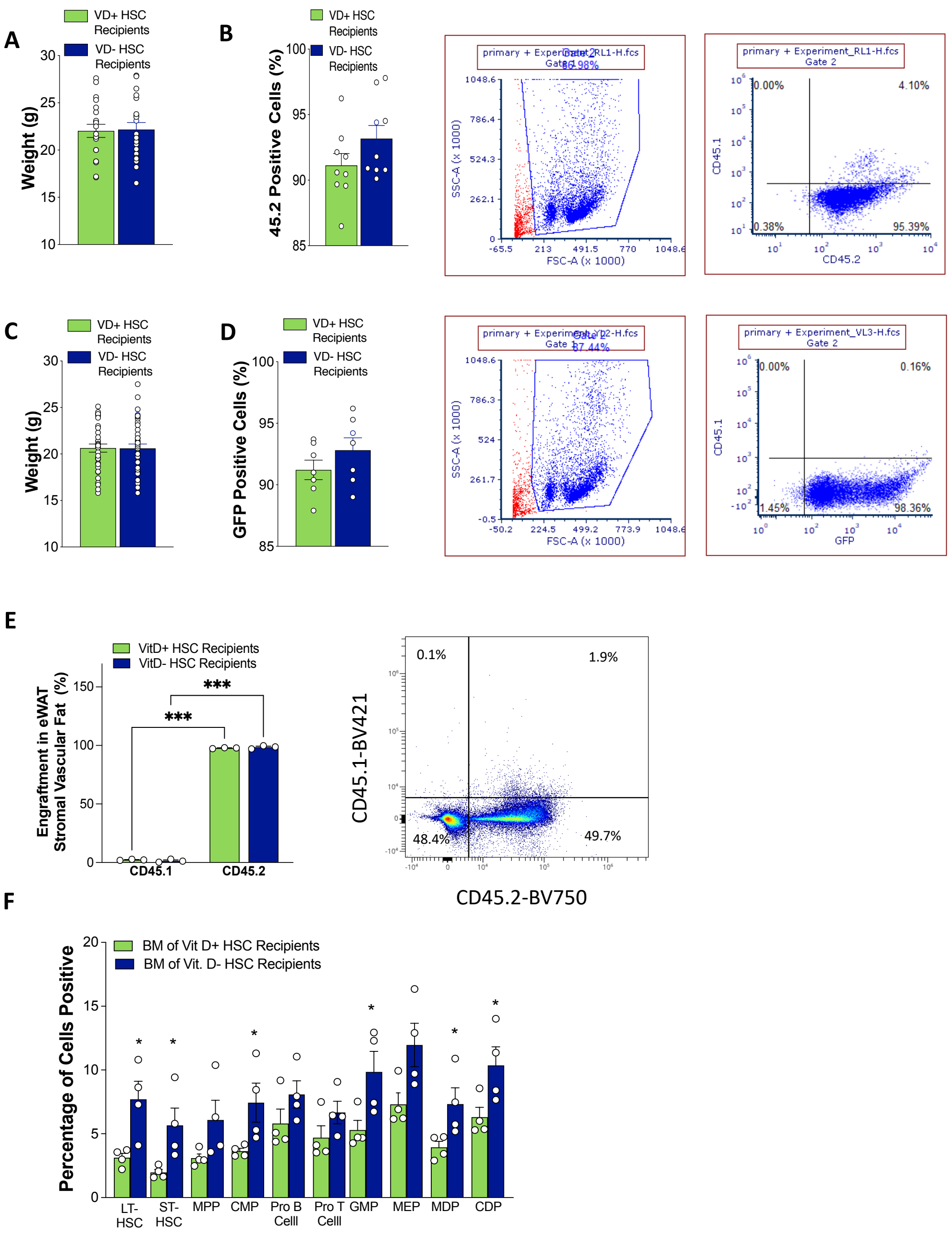

Supplemental Figure 3. Vitamin D status of HSCs does not alter recipient weight or engraftment. HSCs isolated from embryos exposed to in-utero vitamin D sufficiency or deficiency were transplanted into vitamin D-sufficient recipient mice in C57BL6 and LDLR$^{-/-}$ backgrounds. **(A)** Weight (n=20 per group) and **(B)** percentage of circulating CD14^{+} 45.2^{+} cells assessed by flow cytometry (n=9 per group) was measured 8 weeks after transplantation into C57BL6 (CD45.1^{+}) recipient mice. **(C)** Weight (n=35 VD+, n=38 VD-) and **(D)** percentage of circulating GFP^{+} cells (n=7 per group) were measured 8 weeks after transplantation into LDLR$^{-/-}$GFP$^{-/-}$ recipient mice. **(E)** The percentage of 45.1^{+} and 45.2^{+} cells from epididymal stromal vascular fat (SVF) was also measured 30 weeks post-transplant (n=3 per group) in the C57BL6 transplant model. **(F)** Prevalence of immune cell progenitors in BM from primary transplant recipients at 26 weeks in the C57BL6 transplant model (n=4/group). Analyzed as in Fig S2. Data presented as mean ± SEM. *p<0.05, ***p<0.001 by two-tailed unpaired t-test. Actual p values are shown in the source data file.

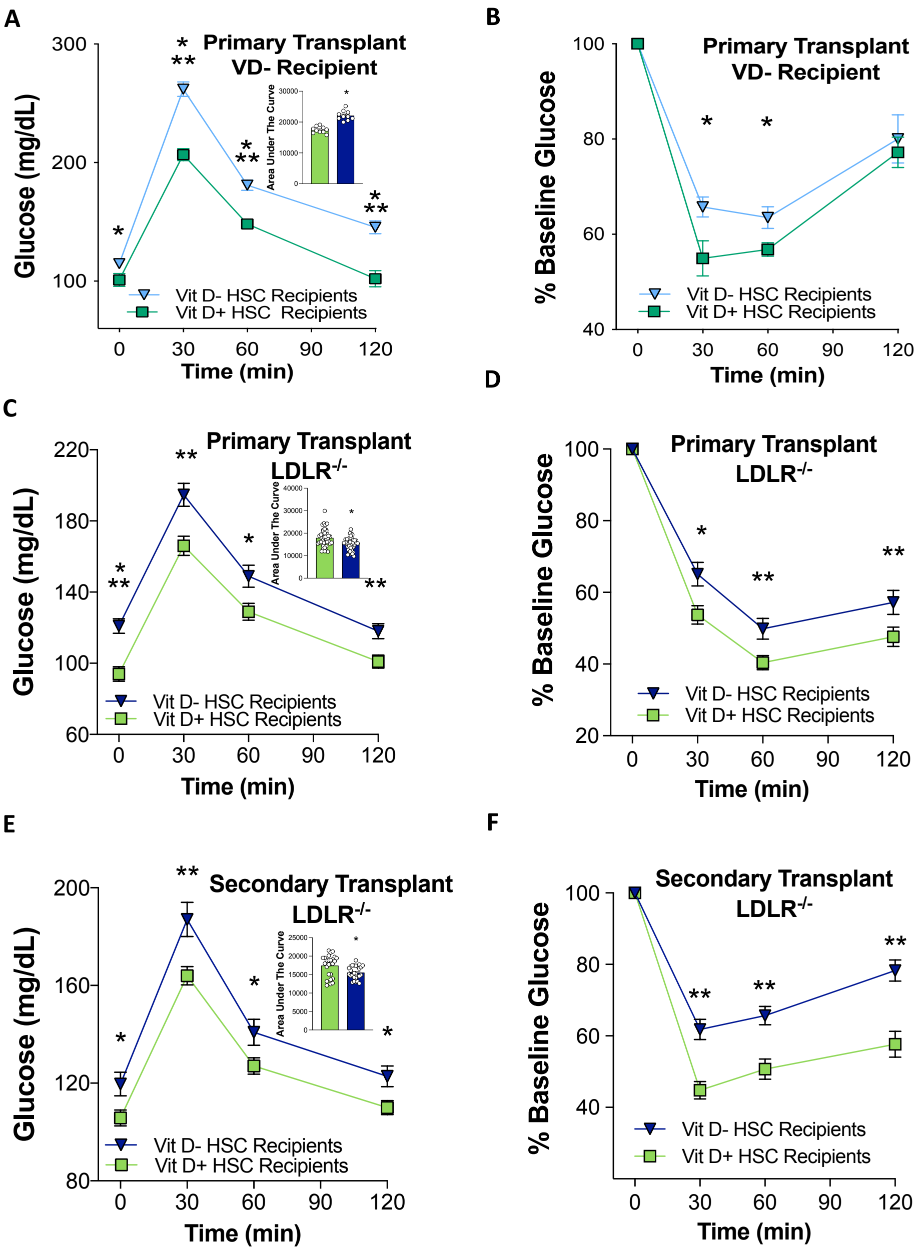

Supplemental Figure 4. In utero, vitamin D deficiency reprograms HSCs to transfer insulin resistance in C57BL6 or LDLR-/- background. (A, B) HSCs isolated from embryos exposed to in-utero vitamin D sufficiency or deficiency were transplanted into vitamin D-deficient recipient mice. Glucose and insulin tolerance tests were performed at (A, B) 8 weeks; n=10/group. (C, D) Vitamin D sufficient LDLR-/- GFP-/- recipient mice were transplanted with VD(-) (n=38) or VD(+) FL-HSCs from LDLR-/- GFP+/- (n=42)(primary transplant). (E, F) Bone marrow from VD(-) (E: n=27 or F: n=21) or VD(+) (E: n=26; F: n=21) FL-HSCs LDLR-/- GFP+/- primary recipient was then used as a transplant donors for vitamin D sufficient mice (secondary transplant). Glucose and insulin tolerance tests were performed at (C, D) 8 weeks post-primary-transplants and (E, F) 8 weeks post-secondary-transplant. Data presented as mean ± SEM. *p<0.05,**p<0.01, ***p<0.001 vs. primary or secondary recipients of VD(+) HSCs by two-tailed unpaired t-test. Actual p values are shown in the source data file.

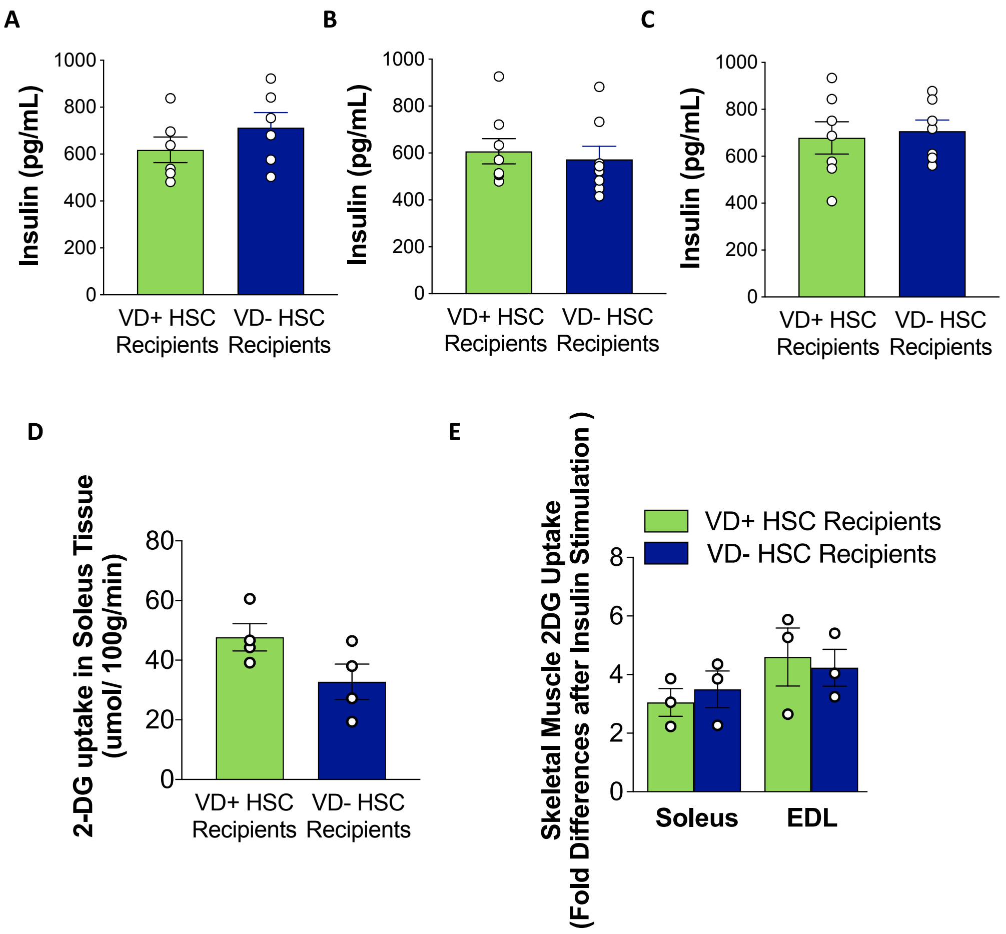

Supplemental Figure 5. Recipient insulin levels after glucose challenge and skeletal muscle insulin resistance are not affected by the vitamin D status of HSCs. Vitamin D sufficient CD45.1^{+} C57BL6 mice were transplanted with VD(-) or VD(+) FL-HSCs from C57BL6 mice CD45.2^{+} (primary transplant). Primary recipient BM was then used as a transplant donor for vitamin D sufficient mice (secondary transplant). Plasma insulin levels were measured 30 min after glucose challenge **(A)** 8 weeks after transplantation (n= 6 per group), **(B)** 6 months after transplantation (n=8 per group), and **(C)** 8 weeks after secondary transplantation (n=7 per group). Skeletal muscle insulin sensitivity was measured in primary recipients 8 weeks post-transplant by **(D)** 2-deoxyglucose (2-DG) uptake in soleus muscle during hyperinsulinemic-euglycemic clamps (n=4 per group) and **(E)** insulin-stimulated 2-DG uptake by ex vivo soleus and extensor digitorum longus muscle (EDL) (n=3 per group). Data presented as mean ± SEM.

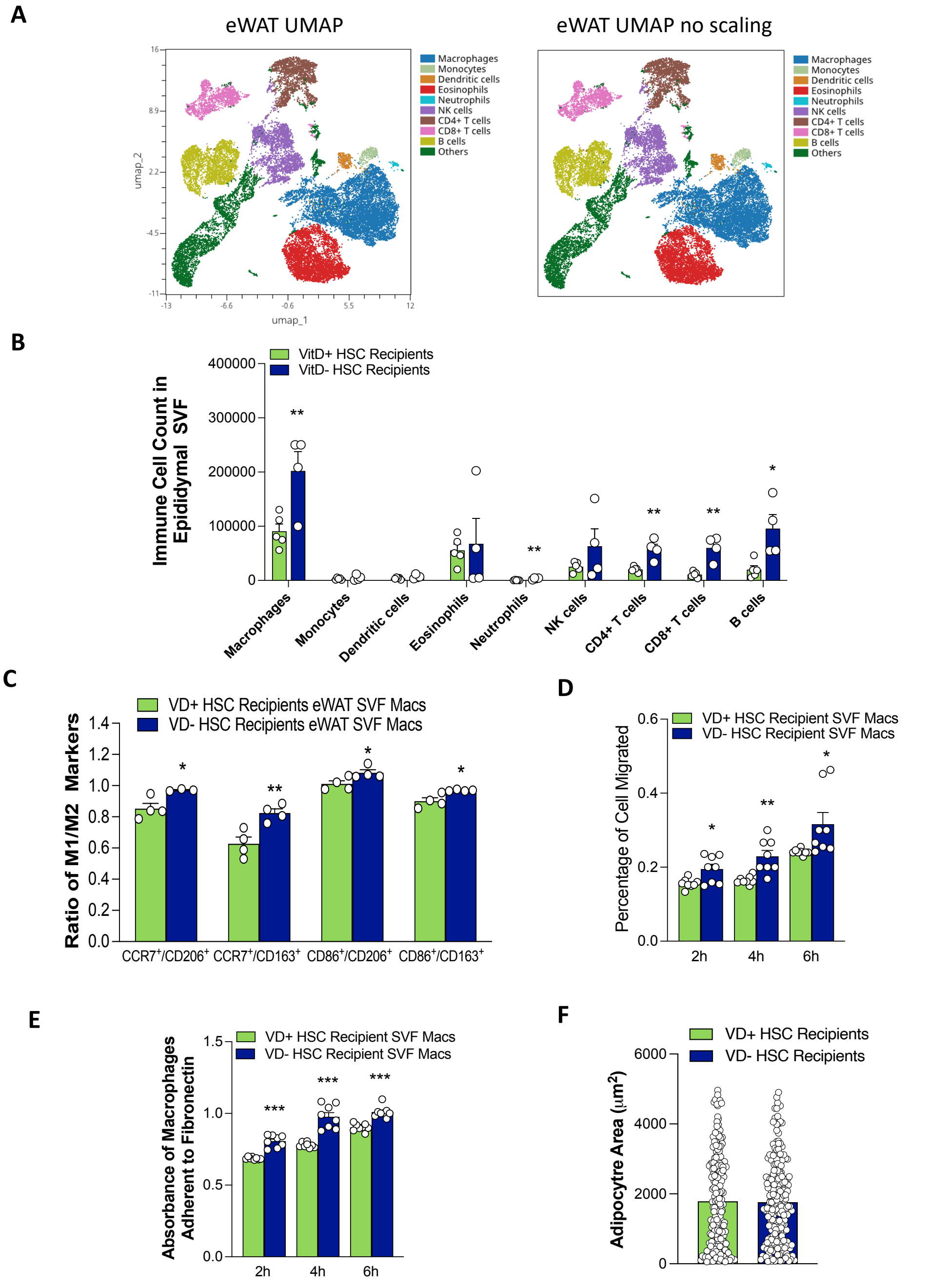

Supplemental Figure 6. Characterization of Immune Cell Infiltration into Tissues. (A-E) Data from Epididymal SVF. (A, B) UMAP visualization of cytometry data from CD45+ eWAT SVF cells (n = 4 per group). Immune cell populations were identified using the FlowSOM algorithm. (B) Quantification of immune cell counts in SVF. (C) Markers of M1(CCR7-CD86)/M2(CD163-206) macrophage phenotype were characterized by flow cytometry (n = 4/group). (D) Transwell migration: percentage of $3x10^5$ $CD14^+$ eWAT SVF macrophages that migrate in response to MCP-1 stimulation (n=8 per group). (E) The absorbance of $CD14^+$ eWAT SVF monocytes adheres to fibronectin (n = 8 per group). (F) Adipocyte size was measured in a minimum of 198 adipocytes from tissue slices from 3 mice/group using ImageJ software. Data presented as mean ± SEM.*p<0.05, **p<0.01, ***p<0.001 by two-tailed unpaired t-test. Actual p values are shown in the source data file.

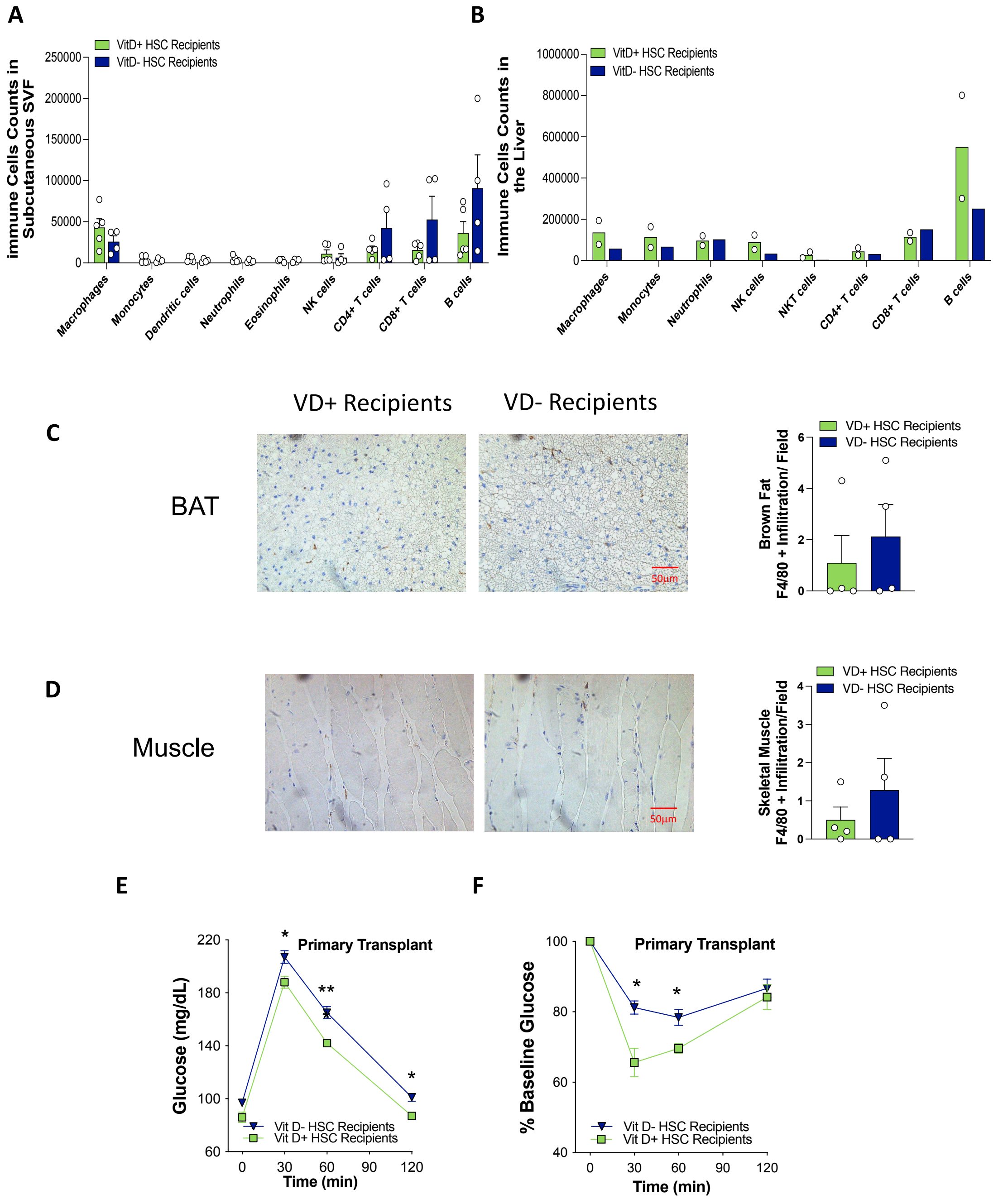

Supplemental Figure 7. Immune Cell Infiltration into Metabolic Tissues. Data from UMAP visualization of cytometry data from CD45.2+ subcutaneous SVF (A) or liver cells (B) (SubCu n = 5/ group; liver n = 2/group). Macrophage infiltration was assessed by F4/80 immunohistochemistry in brown fat (C) and muscle (D). The percentage of F4/80 positive cells was determined by manually counting 15 representative fields under 20x magnification (n=4/group). Representative images are shown at 40x magnification. (E,F) Glucose and insulin tolerance test at 30 weeks post-transplant when tissues were obtained to characterize the immune cells (n=4/group). Data presented as mean ± SEM. *$p<0.05$, **$p<0.01$ by two-tailed unpaired t-test. Actual p values are shown in the source data file.

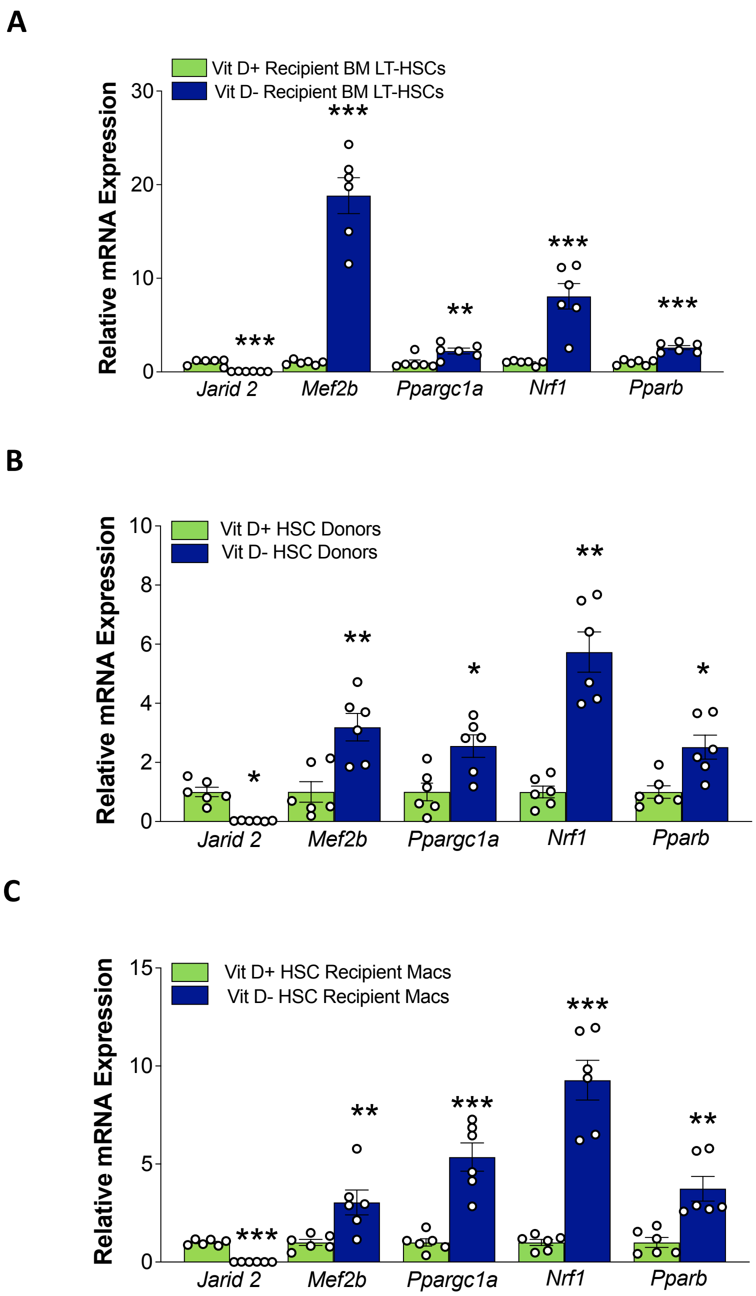

Supplemental Figure 8. In utero, vitamin D deficiency activates the Jarid2/Mef2/PGC1α network in immune cells. Quantitative RT-PCR of target genes of the Jarid2 and PGC1α network in (A) recipient BM LT-HSCs, (n=4/group), (B) LT-HSC donor cells, (n=4/group) and (C) in peritoneal macrophages from FL-HSC transplant recipients (n=6/group). Data presented as mean ± SEM. *p<0.05, **p<0.01, ***p<0.001 by two-tailed unpaired t-test. Actual p values are shown in the source data file.

A

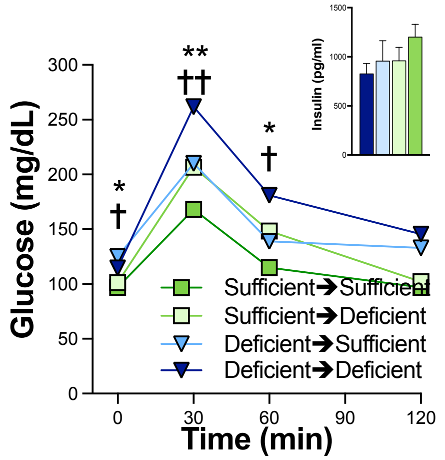

B

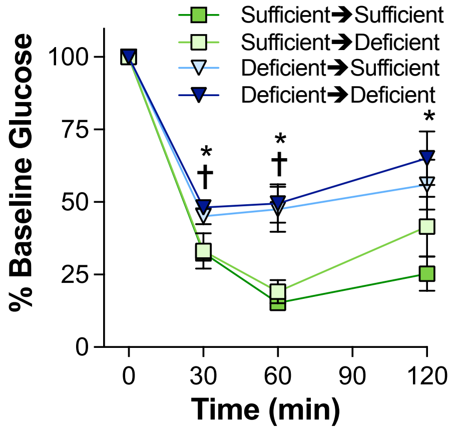

Supplemental Figure 9: VD supplementation after birth does not correct insulin resistance in mice born VD deficient. Offspring born VD deficient or sufficient were transitioned to VD deficient or sufficient diets for eight weeks. (A) Glucose tolerance test performed at 8 weeks (Suff→Suff n= 8, Suff→Def n=16, Def→Suff n=8, and Def→Def n=11). (B) Insulin tolerance test performed at 8 weeks (Suff→Suff n= 7, Suff→Def n=7, Def→Suff n=14, and Def→Def n=19)**.** Data presented as mean ± SEM. *p<0.05, **p<0.01, ***p<0.001 Def→Def vs Suff→Suff VD and †p<0.05; ††p<0.01 for Def→Def vs. Suff→Def by ANOVA followed by Tukey's comparisons. Actual p values are shown in the source data file.

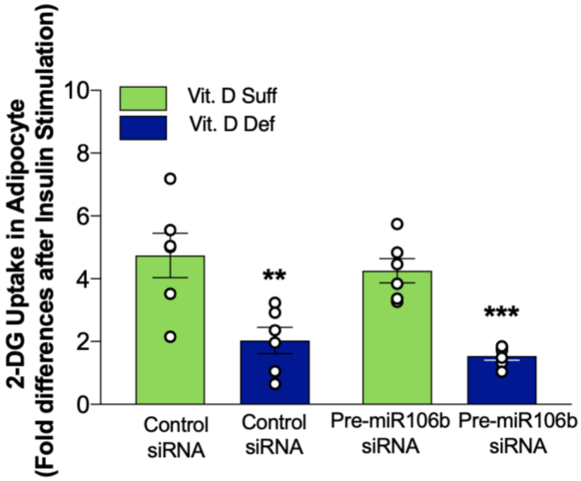

Supplemental Figure 10: Endogenous miR-106b-5p production by the adipocyte does not affect 2-DG uptake. Insulin-stimulated 2-DG uptake in 3T3-L1 adipocytes transfected with premiR-106b-siRNA or control-siRNA and exposed to conditioned media from peritoneal macrophages from VD(+) and VD(-)FL-HSCs recipients (n=6 per group). Data presented as mean ± SEM. **p<0.01, ***p<0.005 by two-tailed unpaired t-test. Actual p values are shown in the source data file.

Enrichr Pathway Analysis

Top Up Regulated Pathways

Term (ESCAPE DATABASE)	Overlap # Genes	P-value	Adjusted P-value
JARID2-20075857 DOWN	42/1107	2.93E-06	9.22E-04
CHiP MYC-18555785	40/1200	9.59E-05	0.015107269
CHiP MYC-19079543	44/1458	3.87E-04	0.040608705
mESC H3K36me3 18692474	66/2469	4.14E-04	0.032576428
CHiP CHD1-19587682	27/843	0.002365341	0.149016486

Term (WikiPathways)	Overlap # Genes	P-value	Adjusted P-value
Macrophage markers WP2271	3/10	7.97E-04	0.140340284
Purine metabolism WP2185	8/171	0.019228259	1
Nucleotide GPCRs WP207	2/12	0.021998268	1
Keap1-Nrf2 WP1245	2/14	0.0295646	1
Adipogenesis genes WP447	6/134	0.047663558	1

Top Down Regulated Pathways

Term (GO Biological Process)	Overlap # Genes	P-value	Adjusted P-value
Viral myocarditis	11/87	1.28E-04	0.03889199
Autoimmune thyroid disease	9/78	0.00101947	0.15445045
Allograft rejection	8/63	0.00102296	0.10331917
Graft-versus-host disease	7/64	0.00490449	0.37151542
Type I diabetes mellitus	7/69	0.00740514	0.44875171
Epstein-Barr virus infection	15/229	0.00892602	0.45076386
Viral carcinogenesis	15/229	0.00892602	0.38636902

Term (KEGG Pathways)	Overlap # Genes	P-value	Adjusted P-value
Positive regulation of stress-activated MAPK cascade (GO:0032874)	9/80	0.00122346	1
Regulation of membrane depolarization (GO:0003254)	4/16	0.00153194	1
Positive regulation of early endosome to late endosome transport (GO:2000643)	3/8	0.00174629	1
Regulation of JNK cascade (GO:0046328)	9/90	0.0027913	1
Positive regulation of JNK cascade (GO:0046330)	8/76	0.00343504	1

Table S1. EnrichR pathway analysis.

Methylation Assay Coordinates

Assay ID	Assay Location	From ATG	From TSS	GRCm38 (+)	# of CpG
ASY3159	5'-Upstream	-2453 to -2351	-1852 to -1750	Chr13:44729419-44729521	6
ASY3160	5'-Upstream	-2211 to -2124	-1610 to -1523	Chr13:44729661-44729748	9
ASY3162	5'-Upstream	-1157 to -1122	-556 to -521	Chr13:44730715-44730750	4
ASY3163	5'-Upstream	-704 to -649	-103 to -48	Chr13:44731168-44731223	6
ASY3164	Exon 1, Intron 1	45 to 89	646 to 690	Chr13:44731916-44731960	2
ASY3166	Intron 1	2149 to 2235	2750 to 2836	Chr13:44734020-44734106	9
ASY3167	Intron 1	35922 to 35937	36523 to 36538	Chr13:44767793-44767808	2
ASY3168	Intron 1	39152 to 39183	39753 to 39784	Chr13:44771023-44771054	3
ASY1677	Intron 1	42317 to 42469	42918 to 43070	Chr13:44774188-44774340	4
ASY1678	Intron 1	42675 to 42679	43276 to 43280	Chr13:44774546-44774550	2
ASY3169	Intron 1	48158	48759	Chr13:44780029	1
ASY3170	Intron 2	108192 to 108219	108793 to 108820	Chr13:44840063-44840090	2
ASY3173	Intron 2	110157 to 110181	110758 to 110782	Chr13:44842028-44842052	2
ASY3175	Intron 2	112064 to 112117	112665 to 112718	Chr13:44843935-44843988	3
ASY3176	Intron 3	119607 to 119651	120208 to 120252	Chr13:44851478-44851522	2
ASY3178	Intron 3	125037 to 125120	125638 to 125721	Chr13:44856908-44856991	3
ASY3179	Intron 4	148554	149155	Chr13:44880425	1
ASY3180	Intron 5	158766	159367	Chr13:44890637	1
ASY3181	Intron 5	159670 to 159719	160271 to 160320	Chr13:44891541-44891590	2
ASY3184	Intron 7	172605	173206	Chr13:44904476	1
ASY3185	Intron 8	174879 to 174908	175480 to 175509	Chr13:44906750-44906779	2
ASY3186	Intron 9	176115 to 176136	176716 to 176737	Chr13:44907986-44908007	2
ASY3188	Exon 10	178525 to 178563	179126 to 179164	Chr13:44910396-44910434	3
ASY3190	3'-UTR	188691 to 188728	189292 to 189329	Chr13:44920562-44920599	3

Table S2. Next-generation sequencing methylation assay coordinates.

List of down regulated microRNAs in Recipients Bone Marrow Cells

Gene Symbol	Mean expression recipients of VD- FL-HSC	Mean expression recipients of VD+ FL-HSC	Ratio Recipient VD- FL-HSC /VD+ FL-HSC	P Value
Mir19b-1	8.35	14.53	0.57	2.64E-03
Mir106b	9.83	14.60	0.67	1.12E-02
Mir142	80.10	111.27	0.72	2.85E-02
Mir331	9.85	13.76	0.72	2.21E-02
Mirlet7g	13.20	18.00	0.73	1.76E-02
Mir340	23.68	32.16	0.74	1.29E-02
Mir330	32.60	41.23	0.79	4.36E-03
Mir376b	3.49	4.33	0.80	2.72E-02
Mir666	53.52	58.36	0.92	3.02E-02
Mir679	25.32	28.72	0.88	3.32E-02
Mir467b	3.49	3.85	0.91	3.66E-02
Mir125a	24.14	27.91	0.86	4.57E-02
Mir92b	73.49	81.69	0.90	1.08E-02

Table S3. Down-regulate miRNAs from HSC transplant bone marrow.

Demographic Characteristics of Population at Delivery

Maternal Characteristics	(N=30)
Age in years (Mean±SE)	28.4 ± 1.1
African American (%)	50
Caucasian (%)	47
Asian (%)	3
Pre-pregnancy BMI (Mean±SE)	27.7 ± 1.32
BMI At Delivery (Mean±SE)	32.6 ± 1
Vaginal Delivery (%)	53
C-Section Delivery (%)	47
Prenatal Vitamin Supplementation (%)	96%
**Infant Characteristics**	
Gender Females (%)	57
Birth Weight (g) (Mean±SE)	3422 ± 81
Birth Height (cms) (Mean±SE)	50.7 ± 0.36

Table S4. Demographic characteristics of the population at delivery.
