# Supplementary material for: Embryonic vitamin D deficiency programs hematopoietic stem cells to induce type 2 diabetes
Source: Nat Commun. 2023 Jun 13;14:3278. doi: 10.1038/s41467-023-38849-z (PMC10264405; doi:10.1038/s41467-023-38849-z)
Supplement: Supplementary file 2 — Reporting Summary [file 41467_2023_38849_MOESM2_ESM.pdf]

nature portfolio

Corresponding author(s): Carlos Bernal-Mizrachi

Last updated by author(s): Apr 30, 2023

Reporting Summary

Nature Portfolio wishes to improve the reproducibility of the work that we publish. This form provides structure for consistency and transparency in reporting. For further information on Nature Portfolio policies, see our Editorial Policies and the Editorial Policy Checklist.

Statistics

For all statistical analyses, confirm that the following items are present in the figure legend, table legend, main text, or Methods section.

n/a	Confirmed	
☐	☒	The exact sample size (*n*) for each experimental group/condition, given as a discrete number and unit of measurement
☐	☒	A statement on whether measurements were taken from distinct samples or whether the same sample was measured repeatedly
☐	☒	The statistical test(s) used AND whether they are one- or two-sided<br>*Only common tests should be described solely by name; describe more complex techniques in the Methods section.*
☒	☐	A description of all covariates tested
☐	☒	A description of any assumptions or corrections, such as tests of normality and adjustment for multiple comparisons
☐	☒	A full description of the statistical parameters including central tendency (e.g. means) or other basic estimates (e.g. regression coefficient) AND variation (e.g. standard deviation) or associated estimates of uncertainty (e.g. confidence intervals)
☐	☒	For null hypothesis testing, the test statistic (e.g. *F*, *t*, *r*) with confidence intervals, effect sizes, degrees of freedom and *P* value noted<br>*Give P values as exact values whenever suitable.*
☒	☐	For Bayesian analysis, information on the choice of priors and Markov chain Monte Carlo settings
☒	☐	For hierarchical and complex designs, identification of the appropriate level for tests and full reporting of outcomes
☐	☒	Estimates of effect sizes (e.g. Cohen's *d*, Pearson's *r*), indicating how they were calculated

Our web collection on statistics for biologists contains articles on many of the points above.

Software and code

Policy information about availability of computer code

Data collection	No software was used for data collection
Data analysis	Data Analysis. Prism 8 version 8.4.3: The array signal data was processed with Partek Genomics Suite 6.6 (Partek, St Louis, MO): Enrichment pathway analysis using Enrichr, Embryonic Stem Cells Atlas of Pluripotency Evidence (ESCAPE), gene Ontology (GO), Kyoto Encyclopedia of Genes and Genomes (KEGG), and Wiki-pathway databases. Methylation Analysis: FASTQ files from the Ion Torrent S5 server were aligned to the local reference database using open-source Bismark Bisulfite Read Mapper with the Bowtie2 alignment algorithm.

For manuscripts utilizing custom algorithms or software that are central to the research but not yet described in published literature, software must be made available to editors and reviewers. We strongly encourage code deposition in a community repository (e.g. GitHub). See the Nature Portfolio guidelines for submitting code & software for further information.

Data

Policy information about availability of data

All manuscripts must include a data availability statement. This statement should provide the following information, where applicable:
- Accession codes, unique identifiers, or web links for publicly available datasets
- A description of any restrictions on data availability
- For clinical datasets or third party data, please ensure that the statement adheres to our policy

RNA sequences from bone marrow arrays (GSE158763) and mehtylation data (GSE157195) have been deposited in the NCBI GEO repository.

Research involving human participants, their data, or biological material

Policy information about studies with human participants or human data. See also policy information about sex, gender (identity/presentation), and sexual orientation and race, ethnicity and racism.

Reporting on sex and gender	Specimens & data were obtained in a de-identified manner from the Women and Infant Specimen Consortium (WIHSC) IRB#201013004, consisting of 30 healthy pregnant women prior to delivery and their full-term infants. Women with singleton pregnancies resulting in full-term vaginal or C-section delivery were included. Sex from singleton were specified on table 4
Reporting on race, ethnicity, or other socially relevant groupings	Racial identity was provided by the Women and Infant Specimen Consortium (WIHSC) based on skin color.
Population characteristics	Women with singleton pregnancies resulting in full-term vaginal or C-section delivery were included. Intrauterine growth retardation, small/large for gestational age, diabetes (gestational, type 1, and type 2 DM), preeclampsia, chorioamnionitis, acute infection (fever or active herpes), and moderate or severe alcohol or drug abuse during pregnancy were excluded. We included the pregnant mothers' age,race, pre-pregnancy BMI, BMI at delivery, vitamin D levels at delivery and infant birth weight and Height
Recruitment	30 Women with singleton pregnancies resulting in full-term vaginal or C-section delivery that fulfill the inclusion criteria were recruited during the day 7 am to 4pm at Barnes and Jewish hospital by the Women and Infant Specimen Consortium (WIHSC) from 06/26/2017 to 05/15/2018.
Ethics oversight	Washington University Human Research Protection office IRB#201013004

Note that full information on the approval of the study protocol must also be provided in the manuscript.

Field-specific reporting

Please select the one below that is the best fit for your research. If you are not sure, read the appropriate sections before making your selection.

[x] Life sciences [ ] Behavioural & social sciences [ ] Ecological, evolutionary & environmental sciences

For a reference copy of the document with all sections, see nature.com/documents/nr-reporting-summary-flat.pdf

Life sciences study design

All studies must disclose on these points even when the disclosure is negative.

Sample size	Sample size for these experiments was based on the effect size on glucose tolerance test from our previous publication from bone marrow transplants from vitamin D receptor knockdown in macrophages(KODMAC) into WT mice and from WT into KODMAC (Oh J, Riek AE, Darwech I, Funai K, Shao J, Chin K, Sierra OL, Carmeliet G, Ostlund RE Jr, Bernal-Mizrachi C.. Cell Rep. 2015 Mar 24;10(11): 1872-86). Based on these results, 10 animals per group were estimated to give us 90% power to detect a difference between groups at an alpha of 0.05. For subsequent animal experiments of multiple sets of primary transplants and secondary transplants were performed with a target a sample size of 10 mice per group.
Data exclusions	There were no data exclusions.
Replication	All animal glucose and insulin tolerance test measurements consisted of at least 10 mice per group of primary and secondary transplanted mice, they were validated in at least in two sets of mice. For all cellular experiments, assays were carried out in duplicate or triplicate, and each experiment was performed at least twice for replication. All results replicated successfully.
Randomization	Samples and organisms were studies based upon genotyping. Both sexes were included for all experiments, with random allocation based on available animals. Mice within the same experiment were the same age and had the same genetic background.
Blinding	Technicians were blinded to animal assessments because a different technician performed the genotyping prior. Technicians were not blinded to cellular assays but all outcomes were quantitative and objective.

Reporting for specific materials, systems and methods

We require information from authors about some types of materials, experimental systems and methods used in many studies. Here, indicate whether each material, system or method listed is relevant to your study. If you are not sure if a list item applies to your research, read the appropriate section before selecting a response.

Materials & experimental systems

n/a	Involved in the study
☐	☒ Antibodies
☐	☒ Eukaryotic cell lines
☒	☐ Palaeontology and archaeology
☐	☒ Animals and other organisms
☒	☐ Clinical data
☒	☐ Dual use research of concern
☒	☐ Plants

Methods

n/a	Involved in the study
☒	☐ ChIP-seq
☐	☒ Flow cytometry
☒	☐ MRI-based neuroimaging

Antibodies

Antibodies used

Anti-CD11b, AAPC-CD45.1, Anti-mouse PE-CD45.2, Anti- IgG2a/K , Anti-PE mouse IgG2a/K , Anti-Phosphor-Akt ab, Anti-AKT, Anti-F4/80 , Anti-TNF-alpha, Anti-Il-1beta,anti-IL-6, Anti Beta Actin, Rat IgG2a K isotype Control, anti-CD86 eFluor450, anti-CD163-Cy5.5, anti-CD206-Alexa700, Sca1- Ly-6A-Alexa Fluor® 700, CD117-APC, CD150-APC 780, CD34 - PE-Cy7, Flt3 - PE-Cy5, B220-efluor 450, CD43-PE, CD25-PE, CD44-APC, CD16/32-eFluor 450, CD115 APC-eFluor 780, CD45.2 – BV750, CD11b - BV605, CD11c - PE-Cy5, CD3 - BV650, CD4 - PE-Dazzle594, CD8 – PE, CD19 -BV711, NK1.1 - BV480, Ly-6C - BV570, Ly-6G – FITC, Siglec-F - APC-Cy7, FcεRIα - Alexa Fluor® 700, CD45.1 - BV421, anti-PIK3CA, anti-PI3KR1, anti-PDPK1

Validation

Anti-CD11b antibody
Abcam
Catalog # ab8878
https://www.abcam.com/cd11b-antibody-m170-ab8878.html

AAPC-CD45.1
Company Name: BioLegend,
Cat#: 110714
Clone#: A20
Company Validation: https://www.biolegend.com/en-us/products/apc-anti-mouse-cd45-1-antibody-2319
Reference: Phan, Tri Giang, et al. "Subcapsular encounter and complement-dependent transport of immune complexes by lymph node B cells." Nature immunology 8.9 (2007): 992-1000.

anti-mouse PE-CD45.2
Company Name: BioLegend
Cat#: 109808
Clone#: 104
Company Validation: https://www.biolegend.com/en-us/products/pe-anti-mouse-cd45-2-antibody-7
Reference: : Phan, Tri Giang, et al. "Subcapsular encounter and complement-dependent transport of immune complexes by lymph node B cells." Nature immunology 8.9 (2007): 992-1000.

mouse IgG2a/K
Company Name: BioLegend,
Cat#: 400219
Clone#: MOPC-173
Company Validation: https://www.biolegend.com/en-us/products/apc-mouse-igg2a-kappa-isotype-ctrl-1397
Reference: Lewis DR, Petersen LK, York AW, Zablocki KR, Joseph LB, Kholodovych V, Prud'homme RK, Uhrich KE, Moghe PV. Sugar-based amphiphilic nanoparticles arrest atherosclerosis in vivo. Proc Natl Acad Sci U S A. 2015 Mar 3;112(9):2693-8. doi: 10.1073/pnas.1424594112. Epub 2015 Feb 17. PMID: 25691739; PMCID: PMC4352838.

PE mouse IgG2a/K
Company Name: BioLegend,
Cat#: 400211
Clone#: MOPC-173
Company Validation: https://www.biolegend.com/en-us/products/pe-mouse-igg2a-kappa-isotype-ctrl-1401
Reference: Ameres S, Besold K, Plachter B, Moosmann A. CD8 T cell-evasive functions of human cytomegalovirus display pervasive MHC allele specificity, complementarity, and cooperativity. J Immunol. 2014 Jun 15;192(12):5894-905. doi: 10.4049/jimmunol.1302281. Epub 2014 May 7. PMID: 24808364.

Phosphor-Akt ab (Ser 473) dilution 1ug/mL)
Company Name: Cell signaling
Cat#: 4058
Clone#: 193H12
Company Validation: https://www.cellsignal.com/products/primary-antibodies/phospho-akt-ser473-193h12-rabbit-mab/4058
Reference: Inoki K, Li Y, Zhu T, Wu J, Guan KL. TSC2 is phosphorylated and inhibited by Akt and suppresses mTOR signalling. Nat Cell Biol. 2002 Sep;4(9):648-57. doi: 10.1038/ncb839. PMID: 12172553.

AKT (#dilution 0.5 ug/mL)
Company Name: Cell signaling
Cat#: 4691
Clone#: C67E7
Company Validation: https://www.cellsignal.com/products/primary-antibodies/akt-pan-c67e7-rabbit-mab/4691?site-search-type=Products&N=4294956287&Ntt=akt&fromPage=plp
Reference: Inoki K, Li Y, Zhu T, Wu J, Guan KL. TSC2 is phosphorylated and inhibited by Akt and suppresses mTOR signalling. Nat Cell Biol. 2002 Sep;4(9):648-57. doi: 10.1038/ncb839. PMID: 12172553.

b-Actin (#dilution 0.5 ug/mL)
Company Name: Cell signaling
Cat#: 8457
Clone#: D6A8
Company Validation: https://www.cellsignal.com/products/primary-antibodies/b-actin-d6a8-rabbit-mab/8457
Reference: Du J, Wang X, Miereles C, Bailey JL, Debigare R, Zheng B, Price SR, Mitch WE. Activation of caspase-3 is an initial step triggering accelerated muscle proteolysis in catabolic conditions. J Clin Invest. 2004 Jan;113(1):115-23. doi: 10.1172/JCI18330. PMID: 14702115; PMCID: PMC300763.

F4/80 for immunofluorescence
Company Name: abcam
Cat#: ab6640
Clone#: CI:A3-1
Company Validation: https://www.abcam.com/f480-antibody-cia3-1-macrophage-marker-ab6640.html
Reference: Xue N, Zhou Q, Ji M, Jin J, Lai F, Chen J, Zhang M, Jia J, Yang H, Zhang J, Li W, Jiang J, Chen X. Chlorogenic acid inhibits glioblastoma growth through repolarizating macrophage from M2 to M1 phenotype. Sci Rep. 2017 Jan 3;7:39011. doi: 10.1038/srep39011. PMID: 28045028; PMCID: PMC5206721.

TNF-a Antibody
Company Name: Cell signaling
Cat#: 7321
Clone#: D1B4
Company Validation: https://www.cellsignal.com/products/primary-antibodies/human-tnf-a-neutralizing-d1b4-rabbit-mab/7321#:~:text=Product%20Description,their%20binding%20to%20biological%20molecules.&text=Human%20TNF%2D%CE%B1%20Neutralizing%20(D1B4)%20Rabbit%20mAb%20has%20been,L%2D929%20mouse%20fibroblast%20cells.
Reference: Hehlgans T, Pfeffer K. The intriguing biology of the tumour necrosis factor/tumour necrosis factor receptor superfamily: players, rules and the games. Immunology. 2005 May;115(1):1-20. doi: 10.1111/j.1365-2567.2005.02143.x. PMID: 15819693; PMCID: PMC1782125.

IL-1β
Company Name: Thermo Fisher
Cat#: 16-7012-81
Clone#: B122
Company Validation: https://www.thermofisher.com/antibody/product/IL-1-beta-Antibody-clone-B122-Monoclonal/16-7012-81
Reference: Shao L, Zhou HJ, Zhang H, Qin L, Hwa J, Yun Z, Ji W, Min W. SENP1-mediated NEMO deSUMOylation in adipocytes limits inflammatory responses and type-1 diabetes progression. Nat Commun. 2015 Nov 24;6:8917. doi: 10.1038/ncomms9917. PMID: 26596471; PMCID: PMC4662081.

IL-6 abs
Company Name: Thermo Fisher
Cat#: 14-7061-81
Clone#: MP5-20F3
Company Validation: https://www.thermofisher.com/antibody/product/IL-6-Antibody-clone-MP5-20F3-Monoclonal/14-7061-81#:~:text=The%20MP5%2D20F3%20antibody%20is,monocytes%2C%20fibroblasts%20and%20endothelial%20cells.
Reference: Macià A, Vaquero M, Gou-Fàbregas M, Castelblanco E, Valdivielso JM, Anerillas C, Mauricio D, Matias-Guiu X, Ribera J, Encinas M. Sprouty1 induces a senescence-associated secretory phenotype by regulating NFκB activity: implications for tumorigenesis. Cell Death Differ. 2014 Feb;21(2):333-43. doi: 10.1038/cdd.2013.161. Epub 2013 Nov 22. PMID: 24270409; PMCID: PMC3890957.

Rat IgG2a K isotype Control
Company Name: ebioscience
Catalog #12-4321-80
Company Validation: https://www.thermofisher.com/antibody/product/Rat-IgG2a-kappa-clone-eBR2a-Isotype-Control/12-4321-80

anti-CD86 eFluor450
Company Name: ebioscience
Catalog # 48-0862-82
Company Validation: https://www.thermofisher.com/antibody/product/CD86-B7-2-Antibody-clone-GL1-Monoclonal/48-0862-82

anti-CD163-Cy5.5

Company Name: Bioss
Catalog # bs-2527R-Cy5.5
Company Validation: https://www.biossusa.com/products/bs-2527r-cy5-5

anti-CD206-Alexa700
Company Name: R&D Systems
Catalog # FAB2535N
Company Validation: https://www.rndsystems.com/products/mouse-mmr-cd206-alexa-fluor-700-conjugated-antibody_fab2535n

Sca1- Ly-6A-Alexa Fluor® 700
Company Name: ebioscience
Catalog # 56-5981-82
Company Validation: https://www.thermofisher.com/antibody/product/Ly-6A-E-Sca-1-Antibody-clone-D7-Monoclonal/56-5981-82

CD117-APC
Company Name: ebioscience
Catalog #17-1172-81
Company Validation: https://www.thermofisher.com/antibody/product/CD117-c-Kit-Antibody-clone-ACK2-Monoclonal/17-1172-82

CD150-APC 780
Company Name: ebioscience
Catalog #47-1502-82
Company Validation: https://www.thermofisher.com/antibody/product/CD150-Antibody-clone-mShad150-Monoclonal/47-1502-82

CD34 - PE-Cy7
Company Name: ebioscience
Catalog #25-0349-42
Company Validation: https://www.thermofisher.com/antibody/product/CD34-Antibody-clone-4H11-Monoclonal/25-0349-42

Flt3 - PE-Cy5
Company Name: ebioscience
Catalog #15-1351-82
Company Validation: https://www.thermofisher.com/antibody/product/CD135-Flt3-Antibody-clone-A2F10-Monoclonal/15-1351-82

B220-efluor 450
Company Name: ebioscience
Catalog #48-0452-6B2
Company Validation: https://www.thermofisher.com/antibody/product/CD45R-B220-Antibody-clone-RA3-6B2-Monoclonal/48-0452-82

CD43-PE
Company Name: ebioscience
Catalog #12-0431-82
Company Validation: https://www.thermofisher.com/antibody/product/CD43-Antibody-clone-eBioR2-60-Monoclonal/12-0431-82

pro T cells: CD25-PE
Company Name: ebioscience
Catalog #12-0259-80
Company Validation: https://www.thermofisher.com/antibody/product/CD25-Antibody-clone-BC96-Monoclonal/12-0259-80

CD44-APC
Company Name: ebioscience
Catalog #17-0441-82
Company Validation: https://www.thermofisher.com/antibody/product/CD44-Antibody-clone-IM7-Monoclonal/17-0441-82

CD16/32-eFluor 450
Company Name: ebioscience
Catalog #48-0161-82
Company Validation: https://www.thermofisher.com/antibody/product/CD16-CD32-Antibody-clone-93-Monoclonal/48-0161-82

CD115 APC-eFluor 780
Company Name: ebioscience
Catalog #47-1152-82
Company Validation: https://www.thermofisher.com/antibody/product/CD115-c-fms-Antibody-clone-AFS98-Monoclonal/47-1152-82

CD45.2 – BV750
Company Name: BD Biosciences
Catalog #747251

Company Validation: https://www.bdbiosciences.com/ja-jp/products/reagents/flow-cytometry-reagents/research-reagents/single-color-antibodies-ruo/bv750-mouse-anti-mouse-cd45-2.747251

CD11b - BV605
Company Name: Biolegend
Catalog #101257
Company Validation: https://www.biolegend.com/en-gb/neuroscience-1/brilliant-violet-605-anti-mouse-human-cd11b-antibody-7637?GroupID=BLG10530

CD11c - PE-Cy5
Company Name: Biolegend
Catalog #117316
Company Validation: https://www.biolegend.com/en-ie/products/pe-cyanine5-anti-mouse-cd11c-antibody-3085

CD3 - BV650
Company Name: Biolegend
Catalog #100229
Company Validation: https://www.biolegend.com/nl-nl/products/brilliant-violet-650-anti-mouse-cd3-antibody-7843

CD4 - PE-Dazzle594
Company Name: Biolegend
Catalog #100566
Company Validation: https://www.biolegend.com/en-us/punchout/punchout-products/product-detail/pe-dazzle-594-anti-mouse-cd4-antibody-9845?GroupID=BLG4211

CD8 - PE
Company Name: Biolegend
Catalog #1100708
Company Validation: https://www.biolegend.com/en-us/products/pe-anti-mouse-cd8a-antibody-155

CD19 -BV711
Company Name: Biolegend
Catalog #115555
Company Validation: https://www.biolegend.com/en-us/products/brilliant-violet-711-anti-mouse-cd19-antibody-12075

NK1.1 - BV480
Company Name: BD Biosciences
Catalog #746265
Company Validation: https://www.bdbiosciences.com/en-us/products/reagents/flow-cytometry-reagents/research-reagents/single-color-antibodies-ruo/bv480-mouse-anti-mouse-nk-1-1.746265

Ly-6C - BV570
Company Name: Biolegend
Company Validation: Catalog #128029
https://www.biolegend.com/fr-lu/products/brilliant-violet-570-anti-mouse-ly-6c-antibody-7392

Ly-6G - FITC
Company Name: Biolegend
Catalog #127606
Company Validation: https://www.biolegend.com/fr-lu/products/fitc-anti-mouse-ly-6g-antibody-4775

Siglec-F - APC-Cy7
Company Name: BD Biosciences
Catalog #565527
Company Validation: https://www.bdbiosciences.com/en-us/products/reagents/flow-cytometry-reagents/research-reagents/single-color-antibodies-ruo/apc-cy-7-rat-anti-mouse-siglec-f.565527

FcεRIα - Alexa Fluor® 700
Company Name: Biolegend
Catalog #134324
Company Validation: https://www.biolegend.com/en-us/products/alexa-fluor-700-anti-mouse-fcepsilonrialpha-antibody-12817

CD45.1 - BV421
Company Name: Biolegend
Catalog #110732
Company Validation: https://www.biolegend.com/en-us/products/brilliant-violet-421-anti-mouse-cd45-1-antibody-7255

anti-PIK3CA
Company Name: Abcam

Catalog #ab40776
Company Validation: https://www.abcam.com/pi-3-kinase-catalytic-subunit-alphapik3ca-antibody-ep383y-ab40776.html

anti-PI3KR1
Company Name: Abcam
Catalog #ab191606
Company Validation: https://www.abcam.com/pi-3-kinase-p85-alpha-antibody-epr18702-ab191606.html

anti-PDPK1
Company Name: Abcam
Catalog #ab52893
Company Validation: https://www.abcam.com/pdpk1-antibody-ep569y-ab52893.html

Eukaryotic cell lines

Policy information about cell lines and Sex and Gender in Research

Cell line source(s)	3T3-L1 adipocytes (American Type Culture Collection) CL-173
Authentication	Fully differentiated adipocytes (12 days post differentiation induction) were test by Oil red O staining for neutral fat deposition before any co-culture experiments
Mycoplasma contamination	Cells were not tested for mycoplasma contamination
Commonly misidentified lines (See ICLAC register)	N/A

Animals and other research organisms

Policy information about studies involving animals; ARRIVE guidelines recommended for reporting animal research, and Sex and Gender in Research

Laboratory animals	1. Ldlr KO (B6.129S7-Ldlrtm1Her/J), Jackson Laboratory Stock No:002207<br>2. C57BL/6J, CD45.2, Jackson Laboratory. Stock No: 000664<br>3. C57BL/6 CD45.1, Jackson Laboratory. Stock No: 002014<br>4.C57BL/6 GFP+/- Jackson Laboratory. Stock No:004353<br>5. miR-106b KO (Mirc3tm1.1Tyj/J) Jackson Laboratory stock No 008460<br>6. Vav1-Cre CD45.2+ Jackson Laboratory stock No 00861<br>7. Jarid2fl/fl CD45.2+ Jackson Laboratory stock No 031141
Wild animals	N/A
Reporting on sex	Sex based analysis was considered in the study design. This aggregated sex information is presented for each experiment in the source data file for each individual experiment
Field-collected samples	Mice were housed within a temperature controlled room (21-22 C) under a 12 h light/dark cycle and allowed free access to food and water
Ethics oversight	Protocols were approved by the Washington University Institutional Animal Care and Use Committee (Protocol 21-0127) and complied with ethical regulations for laboratory animal studies.

Note that full information on the approval of the study protocol must also be provided in the manuscript.

Flow Cytometry

Plots

Confirm that:

- [ ] The axis labels state the marker and fluorochrome used (e.g. CD4-FITC).
- [ ] The axis scales are clearly visible. Include numbers along axes only for bottom left plot of group (a 'group' is an analysis of identical markers).
- [ ] All plots are contour plots with outliers or pseudocolor plots.
- [x] A numerical value for number of cells or percentage (with statistics) is provided.

Methodology

Sample preparation	Engraftment verification: Single cell suspensions of blood were stained with fluorochrome-conjugated mAbs (Biolegend,Inc) for surface markers CD45.1 and 45.2. Cell suspensions were fixed with paraformaldehyde and analyzed by Flow cytometry.After isolation, including CD11b selection with microbeads, cells were resuspended in flow cytometry buffer, and

	>105 cells were analyzed for each sample. Monocytes were incubated with 0.2 mg/mL of anti-mouse APC-CD45.1 (BioLegend, #110714) or anti-mouse PE-CD45.2 (BioLegend #109808) for 15 min on ice, then washed before flow cytometry with utilization of APC mouse IgG2a/K (BioLegend #400219) and PE mouse IgG2a/K (BioLegend #400211) isotype controls. EGFP positive cells were excited at 488 nm and measured by flow cytometry at 530 nm.
Instrument	Four-color BD FACScan™ flow cytometer.
Software	Cell aggregates, dead, and cellular debris were excluded based on FSC/SSC. Batch analysis by Flow Jo version 9.6.2 was used for gating
Cell population abundance	Engraftment verification: 100 ul of peripheral blood.
Gating strategy	Cell aggregates, dead, and cellular debris were excluded based on FSC/SCC. Batch analysis by Flow Jo version 9.6.2 was used for gating consistency and selection of positive populations. Unstained samples and blocking with FC were used to decrease autofluorescence and non-specific background. Flow cytometry data is presented as the percentage of fluorophore- or GFP-positive live cells. Also we used a different gating strategy for identification of macrophage dendritic cell progenitors and conventional dendritic cell progenitors. c-Kit+ Flt3+ were also plotted by CD16/32 CD34 identifying megakaryocytes erythroid progenitors; common myeloid progenitors; and granulocyte-macrophage progenitors.

☒ Tick this box to confirm that a figure exemplifying the gating strategy is provided in the Supplementary Information.
